# Linking belowground microbial network changes to different tolerance level towards Verticillium wilt of olive

**DOI:** 10.1186/s40168-020-0787-2

**Published:** 2020-02-01

**Authors:** Antonio J. Fernández-González, Martina Cardoni, Carmen Gómez-Lama Cabanás, Antonio Valverde-Corredor, Pablo J. Villadas, Manuel Fernández-López, Jesús Mercado-Blanco

**Affiliations:** 1 Departamento de Microbiología del Suelo y Sistemas Simbióticos, Estación Experimental del Zaidín, Consejo Superior de Investigaciones Científicas (CSIC), Calle Profesor Albareda 1, 18008 Granada, Spain; 2 Departamento de Protección de Cultivos, Instituto de Agricultura Sostenible, CSIC, Campus ‘Alameda del Obispo’ s/n, Avd. Menéndez Pidal s/n, 14004 Córdoba, Spain

**Keywords:** Microbial functional community, Microbial structural community, *Olea europaea*, Rhizosphere, Root endosphere, *Verticillium dahliae*, Disease tolerance, Co-occurrence networks

## Abstract

**Background:**

Verticillium wilt of olive (VWO) is caused by the soilborne fungal pathogen *Verticillium dahlia*e. One of the best VWO management measures is the use of tolerant/resistant olive cultivars. Knowledge on the olive-associated microbiome and its potential relationship with tolerance to biotic constraints is almost null. The aims of this work are (1) to describe the structure, functionality, and co-occurrence interactions of the belowground (root endosphere and rhizosphere) microbial communities of two olive cultivars qualified as tolerant (Frantoio) and susceptible (Picual) to VWO, and (2) to assess whether these communities contribute to their differential disease susceptibility level*.*

**Results:**

Minor differences in alpha and beta diversities of root-associated microbiota were detected between olive cultivars regardless of whether they were inoculated or not with the defoliating pathotype of *V. dahliae*. Nevertheless, significant differences were found in taxonomic composition of non-inoculated plants’ communities, “Frantoio” showing a higher abundance of beneficial genera in contrast to “Picual” that exhibited major abundance of potential deleterious genera. Upon inoculation with *V. dahliae,* significant changes at taxonomic level were found mostly in Picual plants. Relevant topological alterations were observed in microbial communities’ co-occurrence interactions after inoculation, both at structural and functional level, and in the positive/negative edges ratio. In the root endosphere, Frantoio communities switched to highly connected and low modularized networks, while Picual communities showed a sharply different behavior. In the rhizosphere, *V. dahliae* only irrupted in the microbial networks of Picual plants.

**Conclusions:**

The belowground microbial communities of the two olive cultivars are very similar and pathogen introduction did not provoke significant alterations in their structure and functionality. However, notable differences were found in their networks in response to the inoculation. This phenomenon was more evident in the root endosphere communities. Thus, a correlation between modifications in the microbial networks of this microhabitat and susceptibility/tolerance to a soilborne pathogen was found. Moreover, *V. dahliae* irruption in the Picual microbial networks suggests a stronger impact on the belowground microbial communities of this cultivar upon inoculation. Our results suggest that changes in the co-occurrence interactions may explain, at least partially, the differential VWO susceptibility of the tested olive cultivars.

Video abstract.

## Background

Olive (*Olea europaea* L. subsp. *europaea* var. *europaea*) is one of the most important tree crops in temperate areas worldwide. It constitutes an agro-ecosystem of major relevance for southern Europe (Spain, Greece, and Italy standing out as the major producing countries) where 61.8% of the global olive production is concentrated, followed by Africa (17.9%), Asia (16.9), and the Americas (2.8%) [1]. However, a number of both traditional and emerging pathogens may jeopardize the future of olive cultivation as a strategic commodity, particularly in the Mediterranean Basin. Verticillium wilt of olive (VWO), caused by the soilborne fungus *Verticillium dahliae* Kleb., is considered one of the most devastating maladies in many regions where olive trees are cultivated [2]. In addition, this disease is very difficult to control due to a number of causes comprehensively and critically reviewed elsewhere [2, 3]. Severity of VWO attacks depends, among other epidemiological factors, on the virulence of the *V. dahliae* pathotype that infects the tree. Traditionally, *V. dahliae* isolates infecting olive are classified into defoliating (D) and non-defoliating (ND) pathotypes. The D pathotype (lineage 1A) is highly virulent and usually causes a severe wilting syndrome including chlorosis, fall of green leaves (defoliation), and death of the tree. Overall, D isolates are more virulent than ND isolates and olive cultivars qualified as tolerant to the ND pathotype can be severely affected by representatives of the D pathotype [2–4]. Thus, under current phytopathological scenarios found in many olive-cultivating regions, the implementation of an integrated management strategy of VWO is recommended since no single control measure has proven to be effective when applied individually [2].

The use of tolerant/resistant olive cultivars is considered one of the most efficient control tools. Indeed, cultivated varieties or wild olive genotypes (*O. europaea* subsp. *europaea* var. *sylvestris* Brot.) displaying tolerance (i.e., able to stand *V. dahliae* infections without developing severe disease symptoms in contrast to susceptible cultivars [5]) can be used to substitute dead trees, as rootstocks, or as source of VWO resistance in breeding programs [6, 7]. Unfortunately, no olive cultivar has so far been reported as fully resistant to VWO*.* Studies on tolerance/resistance to VWO have mostly focused on biochemical and physiological [8, 9] or genetic and full transcriptome [5, 10, 11] responses of olive cultivars displaying differential susceptibility to *V. dahliae*.

While studies on specific beneficial components of the olive-associated microbiota have been conducted, some of them aiming to isolate and characterize biological control agents (BCA) against VWO [12–14], only very few examples are available on whole indigenous olive microbial communities [15, 16] and their potential relationship with susceptibility to biotic constraints [17]. Recently, we described the belowground microbial communities of a range of olive cultivars from different geographical origin grown under the same climatic, agronomical and soil conditions, and in the absence of *V. dahliae* pressure [18].

Plant-associated microbial communities are one of the key determinants for plant health and productivity, aiding in nutrient availability and uptake, enhancing stress tolerance, providing disease resistance, and promoting biodiversity [19, 20]. Interestingly, some plant species harbor similar communities when grown in different soils, while different genotypes or cultivars of the same species can host distinct root microbial communities, highlighting the fact that the plant genotype is crucial to shape the composition of its root-associated microbiome [18, 21–23]. Plants select and shape the belowground microbiome, stimulating or repressing certain members of the indigenous microbial communities which can act as the first defense line against soilborne pathogens through a range of mechanisms [2, 20]. The structure, composition and functionality of the root-associated microbiome are not only influenced by the genotype, fitness, and phenology of the host plant but also by the soil’s health. For instance, bacterial and fungal communities of healthy tobacco soils have recently been shown to greatly differ from bacterial wilt (*Ralstonia solanacearum*) infested soils, leading to the hypothesis that healthy soils harbor higher abundance of beneficial microbes thereby improving soil nutrients, plant growth, and control of soilborne diseases [24].

Microbial communities are complex and consist of many taxa potentially interacting among them. The functional competence of a microbial community is thus not equal to the sum of its individual components [25]. Within these communities, microorganisms can engage a large variety of relationships: positive (e.g., cooperating to build up a biofilm that confers antibiotic resistance to its members [26]), negative (e.g., antibiosis or competition for resources [27]), or neutral [26]. The in-depth analyses of associations established among microorganisms may help to identify their environmental niches, reveal their functional roles within communities [28, 29] and determine the ecosystem functioning/stability [30].

Network analysis is a useful tool to explore the mathematical, statistical, and structural properties of a set of items (e.g., microorganisms) and the connections among them [30]. A new approach based on evaluation of the co-abundance among taxa, highlighting the positive and negative biological relationships, has recently been applied to investigate co-occurrence patterns between microorganisms in complex environments, from the human gut to oceans and soils [26]. Co-occurrence patterns are ubiquitous, might be caused by species or genes performing similar or complementary functions, or shared environmental conditions in which microbial species coexist [27, 28, 31]. Bioinformatic network- and co-occurrence analyses give us an idea about the complexity of microbial interaction patterns [26, 30] but they are not suitable to unravel the nature of these interactions. Despite this limitation, analysis of microbial networks is thus important tools for hypothesis. The existence of specific types of microbial interactions and their consequences for population dynamics or functions, however, require testing in relevant model systems. Additionally, technical approaches, such as cross feeding experiments with stable isotopes or fluorescence in situ hybridization and confocal laser scanning microscopy (FISH-CLSM) combined with dual culture assays are extremely useful for testing hypotheses generated in silico [32].

An accurate knowledge on the structure, composition, function, and dynamics of root-associated microbiota of olive cultivars showing differential responses to VWO can help to understand whether and to what extent these microbial communities may contribute to the host tolerance/susceptibility to *V. dahliae.* Moreover, from this basic information more efficient and holistic VWO control approaches (e.g., microbiome-based biocontrol strategies, breeding for resistance considering the associated microbiota of tolerant varieties) within an integrated disease management framework can be envisaged and developed. Therefore, the main objectives of this work were (1) to describe the structure (DNA level) and functionality (RNA level) of the belowground microbial communities (root endosphere and rhizosphere compartments) associated to the olive cultivars Frantoio (VWO-tolerant) and Picual (VWO-susceptible); (2) to evaluate changes in their composition and activity upon inoculation with the highly virulent, D pathotype of *V. dahliae*; and (3) to assess by co-occurrence network analysis possible differential alterations in the root endosphere and rhizosphere microbial interactions of the two olive cultivars due to the presence of *V. dahliae*. The hypotheses to-be-tested were (1) VWO-tolerance level of olive cultivars is related to the differential composition, structure, and functionality (potentially active microorganisms [33]) of their root microbiota, and (2) the presence of *V. dahliae* alters and re-organizes olive root microbial networks what may contribute to the explanation of the cultivar tolerance level to VWO.

## Results

### General characteristics of sequencing datasets

A total of 7,749,457 (bacterial) and 6,919,278 (fungal) raw reads were obtained by high-throughput sequencing of all samples. Only 4,189,961 (bacterial) and 4,829,128 (fungal) good quality reads were finally retained after the clustering. To avoid an overestimation of the diversity, the operational taxonomic units (OTU) with less than 0.005% of the high-quality reads were discarded. Therefore, a total of 1437 bacterial OTUs and 504 fungal OTUs were eventually considered. For alpha diversity comparison, rarefaction was separately performed to the smallest sample of each domain (bacteria and fungi), each kind of nucleic acid (DNA and RNA) and each compartment (rhizosphere and root endosphere). Finally, 443 out of 448 samples (see Additional file 1: Table S1) with a Good’s coverage > 96.64% were retained for downstream analyses.

### Unraveling the belowground microbiota of “Picual” and “Frantoio” prior to the inoculation with *Verticillium dahliae*

Comparing richness (Observed OTUs) and Inverse of Simpson (InvSimpson) alpha diversity index in both microhabitats (root endosphere and rhizosphere), rhizosphere datasets showed higher values than those from the root endosphere. Kruskal-Wallis test for alpha diversity indices showed significant differences between bacterial communities but not between fungal communities (Table 1). When comparing datasets of the same microhabitat but from different communities (i.e., structural [DNA] versus functional [RNA]), significant differences were found in most of the cases except for the alpha diversity index comparison between microbial communities (both bacteria and fungi) from the root endosphere. Concerning the olive cultivars under examination (Picual and Frantoio), no differences were observed in any domain but for significant richness and alpha diversity increases in functional (RNA) rhizosphere bacterial communities of Picual plants (Table 1). A few more differences were detected when comparing each dataset during the time-course of the experiment. However, no differences were found between cultivars when samples were compared at each time-point (data not shown). Concerning beta diversity and focusing on microbial community dynamics (i.e., changes in OTUs relative abundance profiles along time), the major difference was only found between the initial time (T0) and the rest of time-points analyzed (8, 15, and 30 days), regardless of the cultivar, the microhabitat, the nucleic acid or the microbial domain compared. The only exception was the structural (DNA) root endosphere community of Frantoio plants (Table 2). Furthermore, there was hardly any difference when comparing bacterial communities of each cultivar at different time-points. In contrast, a significant difference between fungal communities present in the root endosphere of Picual and Frantoio plants was observed. This difference remained constant over time (Table 2).

**Table 1:** *p* values of alpha diversity indices reveal significant microhabitat-specific bacterial community differences in greenhouse-grown olive cultivars

		Observed OTUs		Inverse of Simpson	
Dataset	Comparison	Bacteria	Fungi	Bacteria	Fungi
Whole	Endo vs Rhizo	***< 0.001***	0.684	***< 0.001***	0.640
Root Endosphere	DNA vs RNA	***0.006***	***0.032***	0.649	0.350
Rhizosphere		***< 0.001***	0.057	***< 0.001***	***0.005***
EndoDNA	Frantoio vs Picual	0.262	0.390	0.233	0.429
EndoRNA		0.270	0.052	***0.003***	0.085
RhizoDNA		0.863	0.372	0.694	0.079
RhizoRNA		***0.015***	0.395	***0.010***	0.077
EndoDNAFra	Control vs Inoculated	0.317	0.290	0.268	***0.045***
EndoRNAFra		***0.034***	0.420	0.220	0.810
RhizoDNAFra		0.471	0.180	0.810	0.430
RhizoRNAFra		0.531	0.420	0.400	0.450
EndoDNAPic		0.420	0.210	0.690	0.540
EndoRNAPic		0.201	0.850	0.400	0.880
RhizoDNAPic		0.306	0.440	0.400	0.690
RhizoRNAPic		0.200	0.360	0.400	0.730

Figures in boldface and italics show significant *p* values (<0.05). *p* values from Kruskal-Wallis tests are shown. *Endo* root endosphere, *Rhizo* Rhizosphere, *Fra* Frantoio, *Pic* Picual, *C* control, *Ino* inoculated with *Verticillium dahliae*

**Table 2:** PERMANOVAs of quantitative beta diversity index show similar microbial communities between olive cultivars and treatments

		Bray-Curtis*	
Dataset	Comparison	Bacteria	Fungi
Whole	Endo *vs.* Rhizo	***0.001***	***0.001***
Root Endosphere	DNA *vs.* RNA	***0.001***	***0.001***
Rhizosphere		***0.001***	***0.001***
EndoDNAFraC	Time course (T0 *vs.* T8 *vs.* T15 *vs.* T30)	0.308	0.109
EndoRNAFraC		***0.019***	***0.011***
RhizoDNaFraC		***0.001***	***0.001***
RhizoRNAFraC		***0.001***	***0.001***
EndoDNaPicC		***0.003***	***0.002***
EndoRNAPicC		***0.001***	***0.001***
RhizoDNAPicC		***0.001***	***0.001***
RhizoRNAPicC		***0.001***	***0.001***
EndoDNA_0	Frantoio *vs.* Picual (each time)	0.163	***0.027***
EndoRNA_0		0.171	***0.041***
RhizoDNA_0		0.064	0.091
RhizoRNA_0		***0.030***	0.104
EndoDNA_8		0.119	***0.018***
EndoRNA_8		0.058	0.271
RhizoDNA_8		***0.008***	***0.028***
RhizoRNA_8		0.091	0.074
EndoDNA_15		0.325	***0.026***
EndoRNA_15		0.058	***0.003***
RhizoDNA_15		0.555	0.632
RhizoRNA_15		0.485	0.615
EndoDNA_30		***0.016***	***0.019***
EndoRNA_30		***0.004***	***0.021***
RhizoDNA_30		0.706	***0.009***
RhizoRNA_30		0.438	0.378
EndoDNA_8	Control *vs.* Inoculated (each time)	0.117	0.313
EndoRNA_8		0.113	***0.049***
RhizoDNA_8		0.161	0.124
RhizoRNA_8		0.074	0.075
EndoDNA_15		0.081	0.115
EndoRNA_15		0.241	0.114
RhizoDNA_15		***0.006***	***0.001***
RhizoRNA_15		***0.009***	***0.001***
EndoDNA_30		0.164	0.808
EndoRNA_30		0.569	0.666
RhizoDNA_30		0.058	***0.017***
RhizoRNA_30		0.054	0.050

Figures in boldface and italics show significant *p* values (<0.05). *Endo* root endosphere, *Rhizo* Rhizosphere, *Fra* Frantoio, *Pic* Picual, *C* control, *Ino* inoculated with *Verticillium dahliae*, *T0* initial time-point of the experiment. Data collected before inoculation. *T8* eight days after inoculation (dai), *T15* fifteen dai, *T30* thirty dai

*p values from PERMANOVA tests are shown

**Fig. 1:**
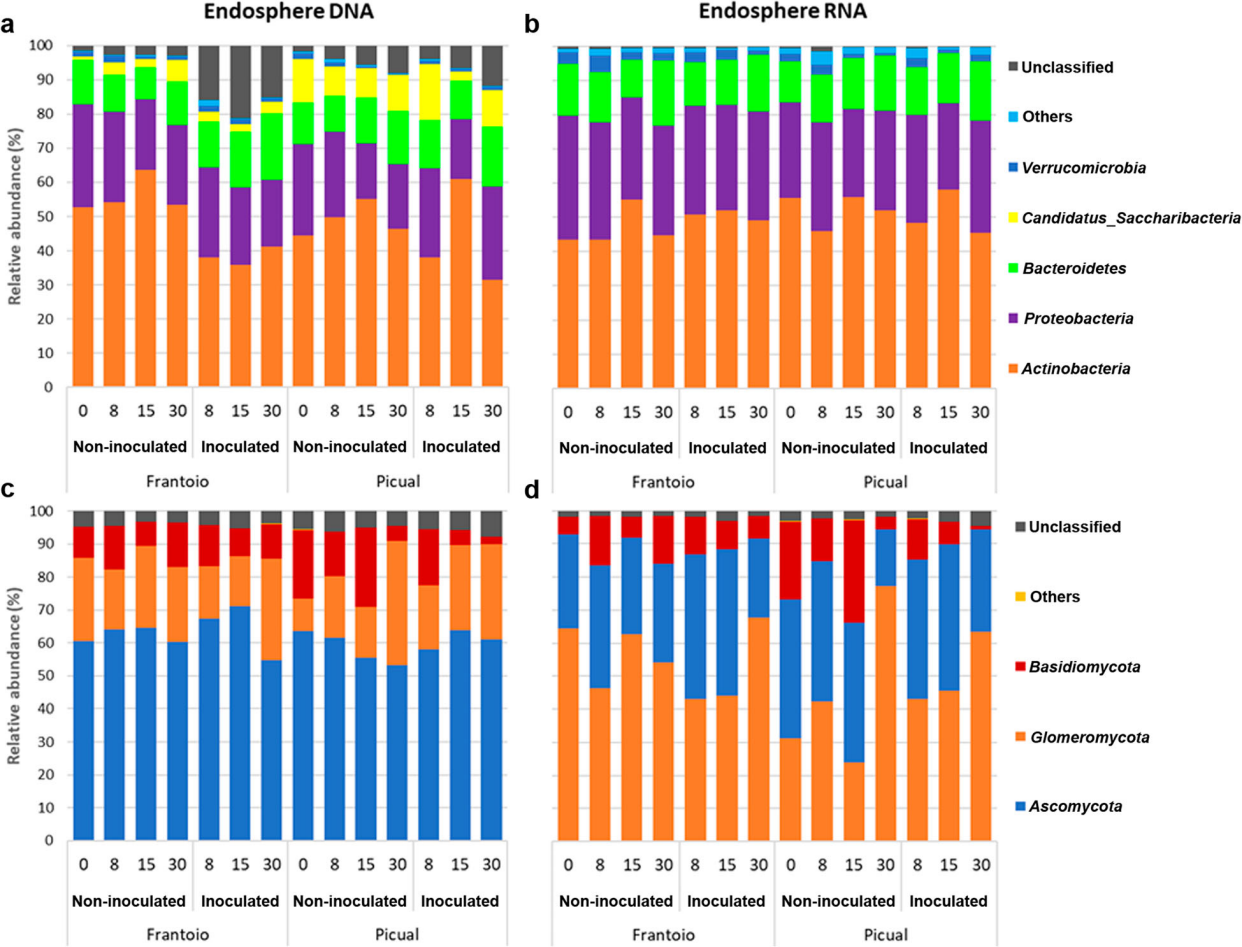
Taxonomical profile at phylum level of the endosphere communities from the studied olive cultivars. **a**, **c** The structural (DNA) communities and **b**, **d** the functional (RNA) communities

Bacterial communities in the root endosphere were dominated by *Actinobacteria*, *Proteobacteria,* and *Bacteroidetes* (74% to 97% of the sequences; Fig. 1a, b)*.* Interestingly, *Candidatus Saccharibacteria* showed a significantly higher (*p* value < 0.01) relative abundance in both structural (DNA) and functional (RNA) communities in Picual than in Frantoio. This phylum had a low relative abundance in functional community and was included in “Others” (Fig. 1b). In contrast, *Proteobacteria* and *Verrucomicrobia* resulted in significantly higher (*p* values, 0.007 and 0.02) relative abundance in the functional community of Frantoio compared to that of Picual. A few differences were found in bacterial community dynamics but they were usually inconsistent (no more than one time-point) or with no clear trend along time (data not shown).

The predominant phyla in fungal communities of the root endosphere were *Ascomycota*, *Glomeromycota,* and *Basidiomycota* (> 90% of the sequences in all samples; Fig. 1c, d). No differences were found between cultivars at any sampling time-point. However, there was a decrease of *Basidiomycota* coupled with an increase of *Glomeromycota* along time (Fig. 1d), although this trend was not statistically significant for any of these phyla. Furthermore, the prevalence of *Ascomycota* and *Glomeromycota* showed an inverted profile when comparing structural (DNA) and functional (RNA) communities (Fig. 1c).

**Fig. 2:**
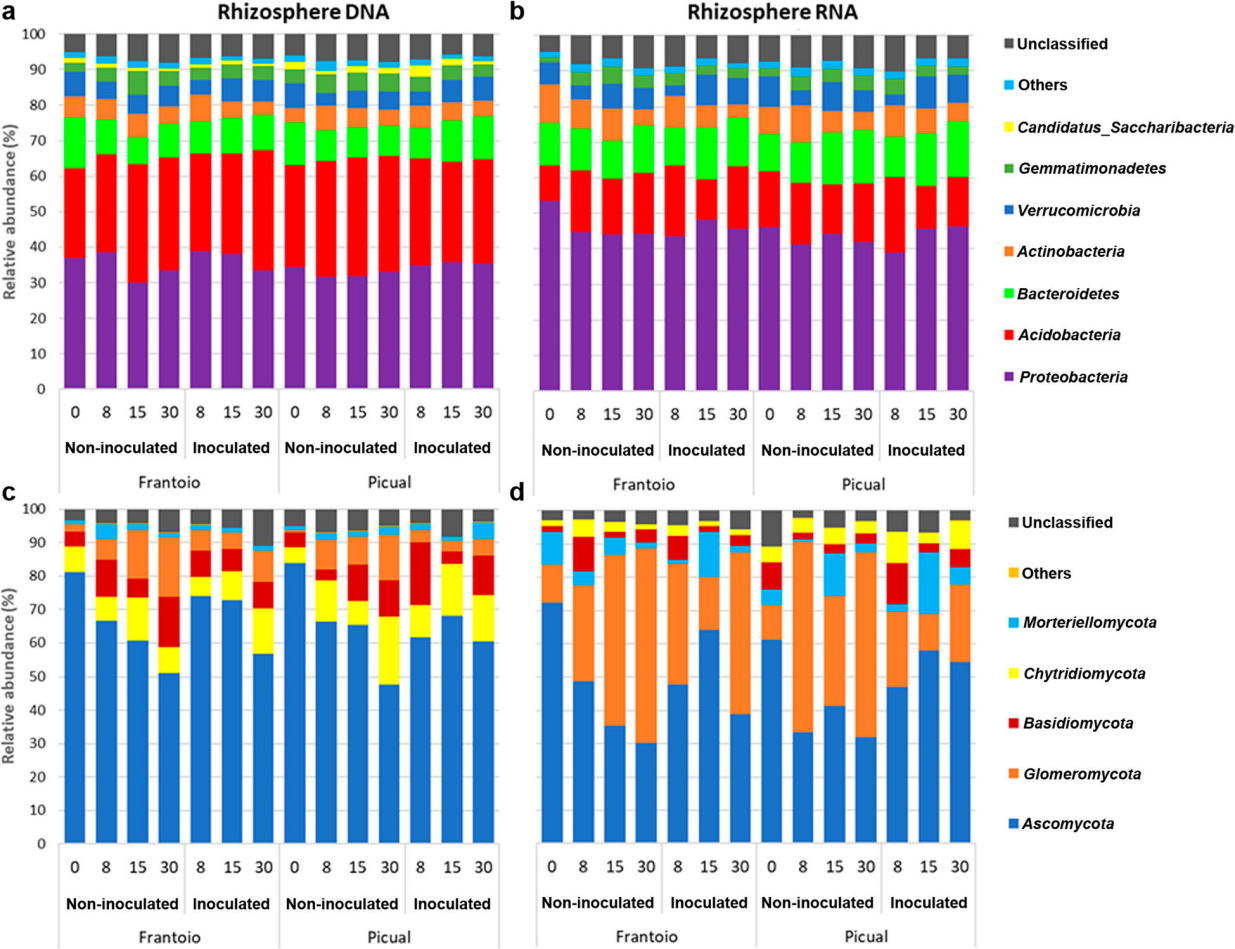
Taxonomical profile at phylum level of the rhizosphere communities from the studied olive cultivars. **a**, **c** show the structural (DNA) communities and **b**, **d** the functional (RNA) communities

Concerning rhizosphere bacterial communities, the predominant phyla were *Proteobacteria* and *Acidobacteria* followed by *Bacteroidetes*, *Actinobacteria*, *Verrucomicrobia,* and *Gemmatimonadetes*, accounting for at least 88% of the sequences (Fig. 2a, b). Likewise to endosphere samples, *Candidatus Saccharibacteria* was significantly higher (*p* value < 0.03) in both structural (DNA) and functional (RNA) communities in Picual than in Frantoio. Furthermore, *Proteobacteria* was also significantly more abundant (*p* value = 0.027) in functional communities of Frantoio than those of Picual. With regard to fungal communities, *Ascomycota* was the predominant phylum in both structural and functional communities. Similarly, to the root endosphere communities, *Glomeromycota* was more abundant in functional than in structural communities. On average, however, this phylum did not overcome *Ascomycota* in this microhabitat (Fig. 2c, d). When comparing functional communities of both cultivars, only *Chytridiomycota* was significantly more abundant in Picual than in Frantoio (*p* value = 0.042) (Fig. 2d).

### Uncovering alterations in the belowground microbiota of olive cultivars upon inoculation with *Verticillium dahliae* D pathotype

No differences in alpha diversity indices were detected when comparing the control, non-inoculated datasets (each cultivar in each microhabitat and for each nucleic acid) versus those ones inoculated with *V. dahliae*. The only exception was a richness decrease in the functional (RNA) bacterial community present in the endosphere of *V. dahliae*-inoculated Frantoio plants. The same was observed for the structural (DNA) fungal community inhabiting the root endosphere of this cultivar (Table 1). Regarding beta diversity, no differences were found in root endosphere microbial communities upon *V. dahliae* inoculation (Table 2). This comparison only showed significant differences (pairwise Adonis *p* value < 0.001) in rhizosphere microbial communities at T15 (15 days after inoculation; dai) in Frantoio but not in Picual plants.

It must be emphasized that since only inconsistent and minor changes were observed in the community dynamics (see previous section), comparisons between microbial profiles of non-inoculated and *V. dahliae*-inoculated plants of both cultivars were performed using the average values of the three sampling time-points chosen in this study (T8, T15, and T30 dai). Concerning endosphere bacterial communities, only *Actinobacteria* in Frantoio (*p* value = 0.007) and *Acidobacteria* (included in Others; Fig. 1a) in Picual (*p* value = 0.028) showed significant differences in relative abundance when comparing non-inoculated versus *V. dahliae*-inoculated samples. This difference was only observed in structural (DNA) communities. Fungal communities showed no significant changes upon inoculation with *V. dahliae*.

Two minor phyla (*Latescibacteria* in DNA and *Candidate division WPS-1* in RNA) were affected in the bacterial communities of the Frantoio rhizosphere upon inoculation with *V. dahliae* (data not shown). In contrast, six phyla were significantly altered in the Picual rhizosphere (structural community), showing a decrease after inoculation with the pathogen, except for *Proteobacteria* (Additional file 2: Figure S1).

With regard to fungal communities, Frantoio samples did not show any noticeable change in the structural (DNA) fungal community of the rhizosphere after inoculation with *V. dahliae* but a decrease in the minor phylum *Mucoromycota* (data not shown). In contrast, the main phylum *Glomeromycota* decreased in both structural (DNA) and functional (RNA) communities (*p* values < 0.03) of Picual samples when the pathogen was introduced in the system (Fig. 2c, d). Indeed, 84.6% of the phylum *Glomeromycota* sequences (arbuscular mycorrhizal fungi, AMF) were classified at the family level*,* and all of them belong to *Glomeraceae*. Interestingly enough, when comparing the root endosphere/rhizosphere ratio of this phylum, a sharp increase was observed in *V. dahliae*-inoculated Picual samples (6.33 in structural and 2.73 in functional communities) compared to the ratio calculated for non-inoculated plants (2.33 and 0.98). In contrast, this ratio increase was clearly moderate in Frantoio samples (from 1.70 in control plants to 3.01 in pathogen-inoculated plants in the structural community, and from 1.18 in control plants to 1.54 in inoculated plants in functional community) (Figs. 1c, d and 2c, d).

### Subtle changes at the genus level in the olive belowground communities

More than 63% of the bacterial sequences were classified at the genus level (> 74% in the case of the root endosphere), ranging from 180 to 188 different genera (Additional file 3: Table S2). Eventually, 83 endosphere and 143 rhizosphere genera were considered as part of the Picual/Frantoio core bacteriome, which represented more than 98% of the classified sequences. The core bacteriome of non-inoculated and *V. dahliae*-inoculated plants shared 100% of the genera (Additional file 4: Table S3).

In non-inoculated plants, 20 and 32 genera showed significant differences (*p* values < 0.05) in relative abundance (average values of the time-points analyzed, i.e., T0, T8, T15, and T30, were used for comparisons) in the root endosphere structural (DNA) and functional (RNA) communities of the two cultivars (Picual versus Frantoio,) respectively. It is worth mentioning that *Cellvibrio* was more abundant in Picual in both communities (DNA and RNA), while *Sphingomonas*, *Pseudonocardia, Bradyrhizobium, Legionella,* and *Rhodanobacter* were more abundant in Frantoio. The rest of the genera showing significant differences were found in just one of these communities (e.g., *Saccharothrix*, *Mycobacterium*, *Streptomyces,* and *Flavobacterium*) or at low relative abundance (less than 0.2%; Additional file 5: Figure S2a, b).

Upon *V. dahliae* inoculation, *Streptomyces* and other 16 genera with very low abundance showed a decrease in Frantoio inoculated plants in the structural (DNA) community of the root endosphere compared to non-inoculated plants (Additional file 6: Figure S3a). In contrast, *Steroidobacter* and *Ohtaekwnagia* increased in this cultivar after pathogen inoculation. In Picual plants, *Gp10* and *Rhodanobacter* experienced a decrease after inoculation, while *Cellvibrio* showed an increase (Additional file 6: Figure S3b).

Less changes were found in the rhizosphere than in the endosphere controls (i.e., 5 genera in structural and 9 in functional communities between Frantoio and Picual plants) (Additional file 7: Figure S4). In the rhizosphere the inoculation with the pathogen only affected few minor genera (relative abundance <0 .2%) in Frantoio plants. Moreover, these genera differed between the structural and functional communities (Additional file 8: Figure S5a, b). A similar pattern was observed in Picual plants with the exception of the main genus *Gp4* that showed a decrease in the structural community of *V. dahliae*-inoculated plants (Additional file 8: Figure S5c, d).

With regard to fungal communities, between 33 and 50% of the sequences were classified at the genus level (ranging from 105 to 129 different genera; Additional file 9: Table S4). Only 17 (root endosphere) and 37 (rhizosphere) genera were considered as part of the shared Picual/Frantoio core mycobiome, representing more than 79% of the classified sequences. As for the core bacteriome, 100% of the core fungal genera were shared in non-inoculated and *V. dahliae*-inoculated plants, but with the expected exception of the genus *Verticillium* which now arose as one of the main genera in the core mycobiome of the rhizosphere of inoculated plants (Additional file 10: Table S5).

In the root endosphere, only 8 (structural community) and 6 (functional community) genera differed significantly when comparing non-inoculated plants of each olive cultivar (Additional file 11: Figure S6a, b). *Macrophomina* and *Fusarium* were more abundant in both structural (DNA) and functional (RNA) communities of Picual control plants, while *Acremonium* and *Lepidosphaeria* were more abundant in Frantoio. Furthermore, *Lophiostoma* and *Rhizoctonia* were more abundant in the Picual structural community, whereas in the case of Frantoio, *Ilyonectrya* was more abundant. Interestingly, *Verticillium* was not found in the root endosphere of pathogen-inoculated plants at any sampling time-point. In summary, no change was observed in this microhabitat as a consequence of *V. dahliae* inoculation.

Concerning the rhizosphere, *Acremonium*, *Lepidosphaeria,* and *Ilyonectria* once again, together with *Chaetomium* and *Cirrenalia*, were significantly more abundant in Frantoio than in non-inoculated Picual plants, both in structural and functional communities (*p* value < 0.05). In contrast, only *Lecanicillium, Plectosphaerella,* and *Setophaeosphaeria* showed significantly higher relative abundance in Picual than in Frantoio (Additional file 12: Figure S7a, b). Upon inoculation with the pathogen, the genus *Gemoyces* increased together with the irruption of *Verticillium* in the rhizosphere of Frantoio plants, while genera *Dominikia* and *Ilyonectria* decreased (Additional file 13: Figure S8a). Finally, in the rhizosphere of pathogen-inoculated Picual plants, besides the appearance of *Verticillium*, genera *Preussia* and *Chaetomium* increased, in contrast to *Fusarium*, *Glomus, Septoglomus,* and *Dominikia* that decreased compared to the situation observed in non-inoculated plants (Additional file 13: Figure S8b).

### Inoculation with *Verticillium dahliae* produces major changes in microbial communities’ network topologies

Co-occurrence networks analysis showed that members of the communities interacted very differently in each microhabitat (root endosphere and rhizosphere). Differences were also found between structural (DNA) and functional (RNA) communities. Interestingly, even though structural and functional communities of each microhabitat were similar (see above), significantly (*p* value < 0.0005) different networks were found between cultivars as a clear effect of the pathogen inoculation (Table 3). For instance, in the presence of *V. dahliae,* the structural community of the Frantoio root endosphere switched to a highly connected (see avgK, GD, and avgCC parameters) and low modularized (see Modularity parameter) network (Table 3). The functional community also showed the same trend although to a lesser extent (Table 3). In contrast, Picual plants showed a sharply different network topology in the root endosphere (i.e., lower connectivity and higher modularization after pathogen inoculation), the functional community displaying more marked changes. However, in the rhizosphere of both cultivars, where the pathogen was present (see above), communities showed similar changes in their network topologies at both structural (DNA) and functional (RNA) levels: decrease of connectivity among nodes, increase of distance between them, and increase of compartmentalization (Modularity). The only difference between structural and functional communities was a decrease of geodesic distance (GD) in the latter one (Table 3). It is worth mentioning that the inoculation with *V. dahliae* increased the number of negative interactions in all cases, with a clearer effect on the endosphere functional communities as revealed by a decrease in positive edge percentage (PEP) (Table 3).

**Table 3:** The major topological properties of Frantoio and Picual co-occurrence networks

Community	No. of original OTUs	Similarity threshold (St)	Total nodes	Total links	Percentage of positive edges (PEP)	R^2^ of power-law	Avg connectivity (avgK)	Avg path distance (GD)	Avg clustering coefficient (avgCC)	Modularity (M)
Fra EndoDNA_C	1299	0.81	278	767	95.44%	0.865	5.518	4.180*	0.253*	0.628 (22)*
Fra EndoDNA_Ino	1227	0.86	334	1669	94.73%	0.819	9.994	3.117*	0.289*	0.418 (37)*
Fra EndoRNA_C	1464	0.77	419	563	78.15%	0.847	2.687	7.051*	0.143*	0.827 (53)*
Fra EndoRNA_Ino	1406	0.87	486	723	66.80%	0.872	2.975	6.323*	0.150*	0.802 (55)*
Pic EndoDNA_C	1332	0.80	280	683	93.41%	0.868	4.879	4.746*	0.258*	0.637 (26)*
Pic EndoDNA_Ino	1149	0.81	337	732	83.61%	0.906	4.344	4.986*	0.248*	0.674 (37)*
Pic EndoRNA_C	1460	0.80	388	805	89.69%	0.944	4.149	4.155*	0.189*	0.609 (49)*
Pic EndoRNA_Ino	1393	0.94	412	503	69.78%	0.925	2.442	8.516*	0.140*	0.854 (51)*
Fra RhizoDNA_C	1850	0.84	530	999	90.89%	0.914	3.770	6.838*	0.179*	0.729 (71)*
Fra RhizoDNA_Ino	1829	0.82	748	871	85.65%	0.881	2.329	8.664*	0.097*	0.893 (136)*
Fra RhizoRNA_C	1871	0.85	616	864	94.21%	0.919	2.805	7.992*	0.174*	0.830 (89)*
Fra RhizoRNA_Ino	1857	0.91	212	172	94.19%	0.849	1.623	3.318*	0.123*	0.911 (70)*
Pic RhizoDNA_C	1850	0.83	595	1397	91.41%	0.891	4.696	5.095*	0.150*	0.608 (86)*
Pic RhizoDNA_Ino	1823	0.83	811	1086	87.48%	0.913	2.678	11.448*	0.157*	0.869 (121)*
Pic RhizoRNA_C	1871	0.84	730	1401	98.29%	0.892	3.838	8.042*	0.224*	0.776 (88)*
Pic RhizoRNA_Ino	1860	0.89	689	1004	88.94%	0.913	2.914	7.815*	0.180*	0.832 (97)*

Significant *p* values (*p* < 0.022) between cultivars are shown in boldface. Asterisks indicate significant *p* values (*p* < 0.0005) between treatments (control versus inoculated). *Pic* Picual, *Fra* Frantoio, *Endo* root endosphere, *Rhizo* rhizosphere, *C* control; *Ino* inoculated with *Verticillium dahliae*. The brackets in Modularity values mean the number of modules in that network

**Fig. 3:**
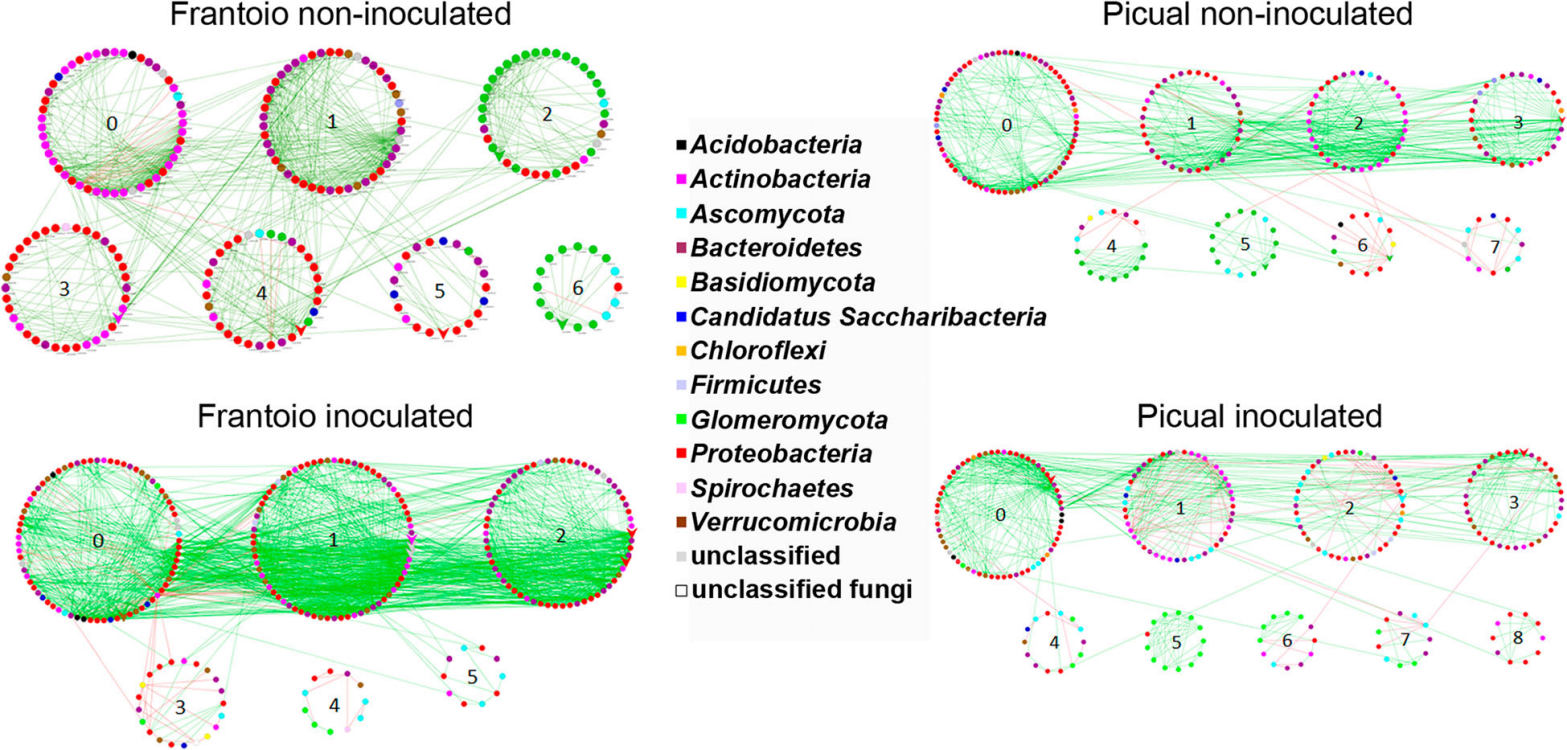
Co-occurrence networks from endosphere structural communities of Frantoio and Picual in control and *Verticillium dahliae*-inoculated plants. Numbers correspond to the number of the module sorted by size

Regarding the root endosphere, both cultivars showed a shift in keystone OTUs after *V. dahliae* inoculation. The most significant change was the lack of *Glomeromycota* in the structural communities of *V. dahliae*-inoculated plants, along with the emergence of some *Ascomycota* in the functional communities (Additional files 14: Figure S9 and Additional files 15: Figure S10). Interestingly, the absence of *Glomeromycota* and the appearance of *Ascomycota* keystone OTUs occurred without the irruption of the pathogen in the network of this microhabitat. The structural and functional community networks of Frantoio plants showed the most noticeable changes due to inoculation with *V. dahliae* (Fig. 3, Additional file 16: Figure S11). The keystone OTU in structural community of non-inoculated control plants classified as *Glomus*, *Rhizophagus* (*Glomeromycota*), *Phenylobacterium*, *Xanthomonas*, *Ferrovibrio*, *Sphingomonas, Gammaproteobacteria* (*Proteobacteria*), *Microbaceteriaceae* (*Actinobacteria*), and *Roseimicrobium* (*Verrucomicrobia*). However, upon inoculation with the pathogen, the former were replaced by *Steroidobacter*, *Rhizobium*, *Pseudomonas*, *Brevundimonas*, *Ancylobacter*, *Legionella*, *Hylemonella* (*Proteobacteria*), *Streptomyces*, *Actinomycetales* (*Actinobacteria*), *Opitutus*, *Prosthecobacter* (*Verrucomicrobia*), *Dyadobacter*, *Chitinophagaceae* (*Bacteroidetes*), and two unclassified *Bacteria*. Only *Devosia* and *Hydrogenophaga* (*Proteobacteria*) were present in both conditions although *Devosia* decreased from three to one representative OTU (Additional file 14: Figure S9). The keystone OTUs for the non-inoculated Picual structural community were *Rhizophagus* (*Glomeromycota*), *Roseimicrobium* (*Verrucomicrobia*), like for Frantoio, *Rhizophagus* (Glomeromycota), *Stenotrophomonas*, *Devosia*, *Steroidobacter*, *Luteimonas*, (*Proteobacteria*), *Actinoplanes* (*Actinobacteria*), and *Bacillus* (*Firmicutes*). After the inoculation all the keystone OTUs were replaced, similarly to Frantoio community, by *Flavobacterium* (*Bacteroidetes*) and *Sphingomonas*, *Rhodanobacter*, *Peredibacter*, *Devosia,* and four unclassified genera, all of them belonging to *Proteobacteria* (Additional file 15: Figure S10).

**Fig. 4:**
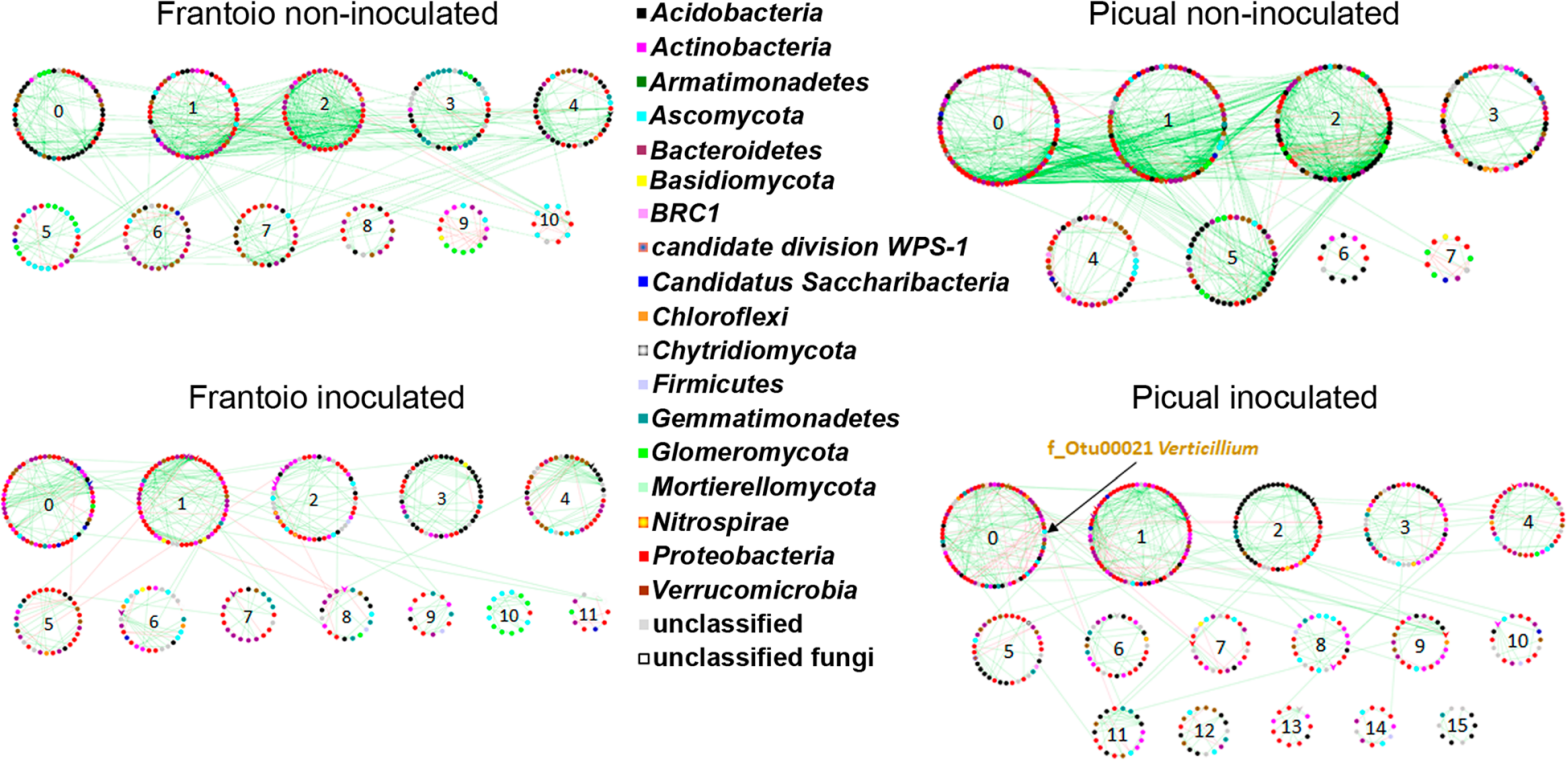
Co-occurrence networks from rhizosphere structural communities of Frantoio and Picual in control and *Verticillium dahliae*-inoculated plants. Numbers correspond to the number of the module sorted by size

Concerning the rhizosphere, co-occurrence networks analysis showed that the structural community of Picual plants experienced the most noticeable changes after inoculation with *V. dahliae*, clearly evidenced by sharp increases of GD and modularity (Table 3). The most obvious change between the two cultivars was the presence of *Verticillium* in Picual networks, at both structural (DNA) and functional (RNA) level. In contrast, this change was not observed in Frantoio networks (Fig. 4, Additional file 17: Figure S12).

## Discussion

The aim of this study was to unravel the structure, functionality, and interactions of rhizosphere and endosphere microbial communities associated with two olive cultivars differing in susceptibility to VWO, and to determine how the introduction of *V. dahliae* can alter them. A first relevant result from our study was that neither the plant genotype nor the inoculation with *V. dahliae* significantly influenced the rhizosphere and endosphere microbial communities of the olive cultivars analyzed, as revealed by the analysis of the alpha and beta diversity .The only constant variation was observed along time between T0 and the other sampling times (T8, T15, and T30 dai), indicating a clear effect after adding either 150 ml of water (control, non-inoculated plants) or 150 ml of a suspension of *V. dahliae* conidia (inoculated plants) on the resident microbial communities.

The most significant alterations were found in the communities’ taxa relative abundances. Comparing the microbial communities of non-inoculated plants of each cultivar, some interesting differences deserve discussion. At phylum level, Picual was always characterized by a higher relative abundance of *Candidatus Saccharibacteria,* both in the rhizosphere and the endosphere and in the structural (DNA) and the functional (RNA) communities, and in contrast to Frantoio plants that showed more abundance in *Proteobacteria*. *Candidatus Saccharibacteria,* formerly known as *Candidate Division TM7,* was recently suggested as a key biomarker in bacterial wilt disease suppression to indicate a state of malady and stress for the plant [34]. This study showed a negative correlation between the relative abundances of *Candidatus Saccharibacteria* and *R. solanacearum* in tobacco plants. In agreement with this finding, we found a decrease in *Candidatus Saccharibacteria* in the rhizosphere structural community of Picual plants (Fig. 2a) after *V. dahliae* inoculation, what could be a consequence of the biotic stress experienced by this cultivar. In contrast, no alteration for this phylum was detected in Frantoio communities. It is tempting to speculate that this phylum may play a role as indicator of stress for Picual upon pathogen inoculation and that this pressure could be related with VWO susceptibility displayed by this cultivar.

We would like to emphasize that in rhizosphere microbial communities and, more importantly, in those of the root endosphere of non-inoculated Frantoio plants, higher relative abundance of genera frequently described as plant growth promoting microorganisms (PGPM) was unveiled, compared to the situation observed in Picual plants. On the one hand, in the rhizosphere bacteriota of the VWO-tolerant cultivar, the genera *Acidovorax* (reported to stimulate growth in barley [35]), *Neorhizobium* (related to hot pepper biomass stimulation [36]), *Nocardia*, *Ancylobacter* (producers of the phytohormone indole-3-acetic acid (IAA) [37, 38]) and *Lentzea* (linked to wheat growth stimulation [39]) must be highlighted. Concerning fungi, the genus *Acremonium* is worth mentioning for its involvement in plant growth [40]. On the other hand, in the Frantoio endosphere, the bacterial genera *Neorhizobium, Bradyrhizobium* (able to form nitrogen fixing nodules in legumes and nodule-like structure in radish, tomato, and rice [41]), *Sphingomonas* (reported to increase Chinese medicinal plant [42] biomass), *Actinoplanes* (involved in cucumber [39] growth promotion), and *Caulobacter* (reported as phytohormones producer in lavender [43]) were significantly more abundant. With regard to fungi and as reported for the rhizosphere, the genera *Trichoderma*, a well-known PGPM and BCA, and *Acremonium* were also found (e.g., [44]). In accordance with Wang et al. [24], who suggest that beneficial microbes harbored in a healthy soil can improve plant growth and control soilborne diseases, we suggest that the higher significant abundance of PGPM in Frantoio microbial community, compared to the situation observed in Picual, could be linked to the VWO tolerance of the former cultivar. On the contrary, Picual endosphere communities were characterized by higher relative abundance of fungi such as *Fusarium*, *Macrophomina,* and *Rhizoctonia*, genera well known for including phytopathogenic species [45–47]. We speculate that the presence of deleterious representatives of these genera could somehow increase the susceptibility of Picual roots to *V. dahliae* attacks*.* Related to this, Khoury and Alcorn [47] reported that the infection by *Rhizoctonia solani* in two varieties of cotton plants induced lesions in the roots, a scenario that could reduce the effectiveness of the physical barriers to hinder colonization by *V. albo-atrum*.

The bacterial root endophytic communities of Picual and Frantoio showed significant changes in some taxa relative abundances, likely explained as a consequence of *V. dahliae* inoculation. For instance, Picual root endosphere showed a decrease in *Acidobacteria*. This agrees with studies suggesting that the presence of this phylum is related to healthy plants, and that its decrease is linked to a diseased state in tobacco infected with *R. solanacearum* [48]. It might well be that the same situation takes place in the VWO-susceptible olive cultivar but not in Frantoio plants. Conversely, a decrease in *Actinobacteria* was observed in the root endosphere of Frantoio (but not in Picual) in contrast to studies reporting that this phylum is responsible of enhanced resistance to *V. dahliae* in tomato [49].

Another interesting result from our study was the changes detected in the rhizosphere communities, the microhabitat where the pathogen firstly enters into direct contact with the plant. Therein, structural and functional microbial communities of Frantoio plants showed significant alterations only in minor phyla of its associated bacteriota and mycobiota, suggesting that the introduction of *V. dahliae* had little effect in the VWO-tolerant cultivar. In contrast, Picual rhizosphere communities underwent major alterations upon pathogen inoculation. Indeed, six phyla showed significant changes in the VWO-susceptible cultivar due to the presence of *V. dahliae*, the decrease of *Gemmatimonadetes* being the most interesting alteration. It is worth mentioning that negative correlation between *Gemmatimonadetes* relative abundance in the rhizosphere and *V. dahliae* infection has been reported in other studies [50]. Inderbitzin et al. [51] also found an increase in *Proteobacteria* after V. *dahliae* infection, in agreement with our results. The activity and positive effect of *Proteobacteria* members on plant health is well documented [19].

Regarding fungi, the Picual rhizosphere showed a significant decrease in *Glomeromycota* relative abundance after inoculation with the pathogen. Genera of this phylum are classified as AMF which are well known to contribute to the health status of the host plant by several modes of action, including the activation of defense mechanism against soilborne pathogens (e.g., *Phytophthora, Fusarium, Verticillium*). The beneficial effects of AMF have been comprehensively reviewed elsewhere [52]. Furthermore, a high ratio between endosphere/rhizosphere *Glomeromycota* relative abundance is in agreement with biomass ratios found in AMF with ruderal strategies, characterized by the capacity to rapidly colonize habitats which are competitor-free due to recent disturbance. Since in our greenhouse experimental conditions neither limiting (e.g., nutrients) nor stressing (e.g., temperature, water) conditions were present, the endosphere/rhizosphere ratios found in non-inoculated plants (and in both cultivars) are in agreement with an early stage of AMF community establishment [53]. However, the increase of this ratio after *V. dahliae* inoculation, with a significant decrease of *Glomeromycota* in Picual rhizosphere, could be attributed to a protective role of AMF. This is in accordance with Newsham et al. [54], who suggested that AMF focused their activity mainly in the root endosphere, playing a defensive role against pathogens rather than acting as nutrient mobilizers. Moreover, the observed AMF endosphere/rhizosphere ratio modification may indicate a state of stress for Picual plants upon pathogen inoculation, partially explaining the higher susceptibility of this cultivar to VWO.

We would like to stress that none of the olive cultivars showed significant differences in the presence/absence of taxa when comparing non-inoculated and *V. dahliae*-inoculated plants. Furthermore, there were no differences at structural and functional levels. Indeed, the bacteriome and mycobiome cores were nearly identical in non-inoculated plants of both cultivars, a similar scenario found between the latter ones and pathogen-inoculated plants. This points to the fact that Frantoio and Picual seem to recruit and harbor similar belowground microbial communities and that the inoculation with *V. dahliae* does not cause noticeable alterations in the diversity of these communities, at least under our experimental conditions.

Network analysis of taxa co-occurrence patterns offers new insights into the structure of complex microbial communities, patterns that otherwise are more difficult to unveil by using the standard alpha/beta diversity metrics widely used in microbial ecology [30]. It has been suggested that complex soil microbial community networks (networks with high number of nodes, number of links, and average connectivity), rather than the simple ones, benefit plants [28]. Indeed, complex networks contribute to better cope with environmental changes or to suppress soilborne pathogens. For instance, tobacco plants associated with rhizosphere microbial communities exhibiting complex networks showed lower incidence of bacterial wilt disease compared to plants associated with communities displaying less connections in their networks [29]. Also, in *Brassica napus* L. seeds microbiome, tightly-knit and complex microbial networks have been observed and proposed as traits that make the invasion by newcomers (either beneficial or pathogenic) of these niches difficult [55]. Our findings are in agreement with these studies. Indeed, the Frantoio endosphere communities showed a marked increase in complexity in the co-occurrence networks after *V. dahliae* inoculation, in contrast to Picual plants (Table 3 and Fig. 3). Co-occurrence interaction studies have been mainly focused on rhizosphere and phyllosphere microhabitats [29, 30]. To the best of our knowledge, we have implemented for the first time this approach to assess a root endosphere community and the topological modifications occurring in this microhabitat upon the introduction of a soilborne pathogen.

A decrease in complexity was observed in the rhizosphere microbial communities of both cultivars after *V. dahliae* inoculation. This change was more pronounced in Picual, particularly in the structural community. Furthermore, a simultaneous increase in the modularity of the rhizosphere microbial networks was also observed in both cultivars. Increase of modularity and GD has been proposed by Delmas et al. [56] as a strategy to maintain the stability of the community, thereby protecting it from disturbances caused by pathogens. Indeed, Cardinale et al*.* [32] have demonstrated an increase in soilborne pathogens biocontrol linked with loose bacterial networks in lettuce roots. Thus, the negative effect of *V. dahliae* would be alleviated by decreasing the interactions (co-occurrence or co-exclusion) between the affected module and the neighboring modules [55]. In principle, rhizosphere microbial communities of both cultivars followed this same strategy, but it was doomed to failure in Picual plants because the pathogen strongly interacted with OTUs of the largest module (Fig. 4 and Additional file 16: Figure S11). This interaction could explain the higher increase in GD observed in the rhizosphere structural community of Picual compared to that of Frantoio. Nevertheless, due to the limitations of the correlation techniques currently available [57], it cannot be completely ruled out that the pathogen might have also interacted with the rhizosphere microbial community of Frantoio plants, a scenario clearly observed in Picual. Finally, our results also indicate that the introduction of *V. dahliae* increases the number of negative interactions (e.g., competition and antagonism) to a larger extent in the VWO-susceptible cultivar (Picual) than in the tolerant cultivar (Frantoio).

## Conclusions

**Fig. 5:**
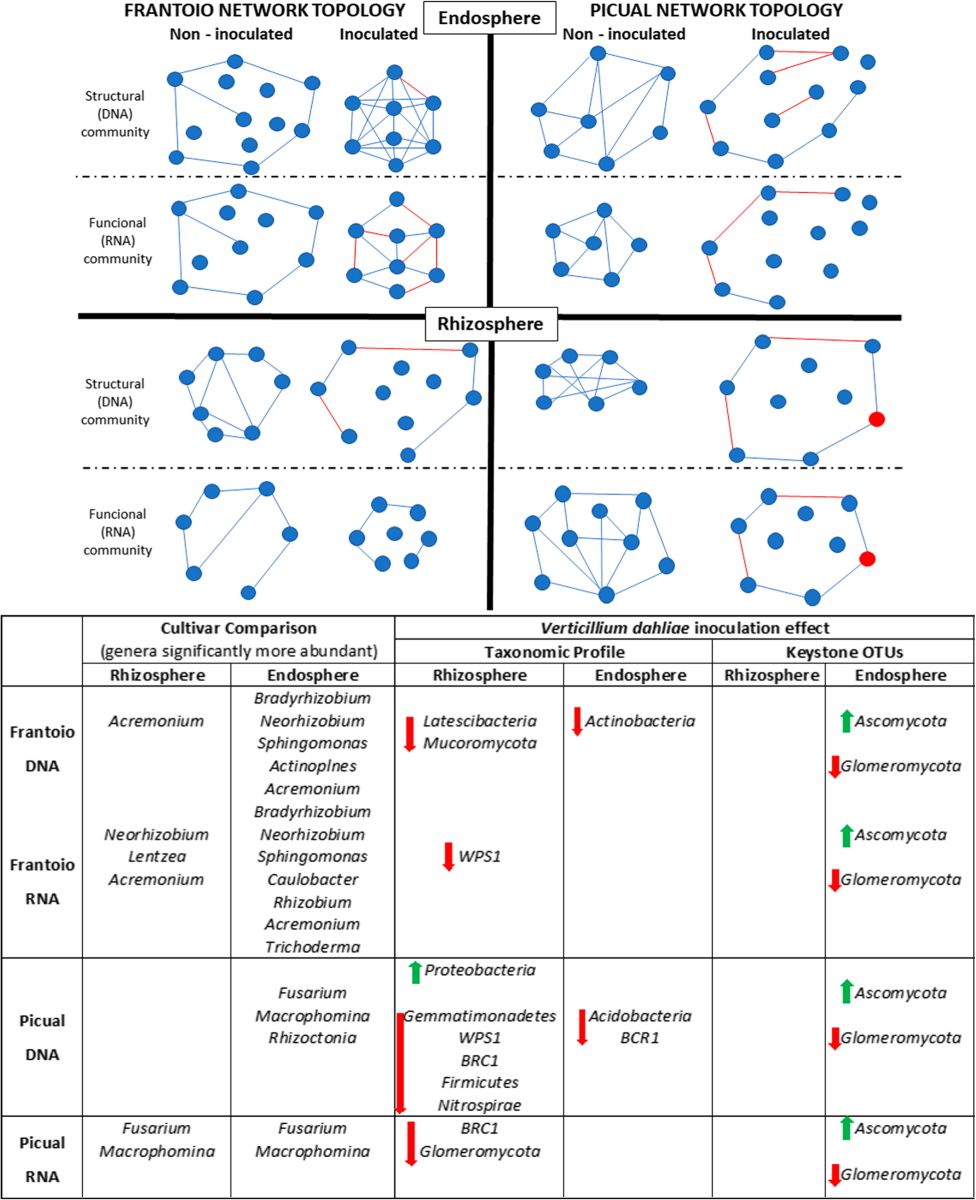
Schematic representation of major findings related to olive cultivars comparison of their belowground microbiome and the effects of *Verticillium dahliae* inoculation in microbial networks. In the network, red edges represent negative interactions between modules (solid circles). The red circle represents the module that includes *V. dahliae*. In the bottom table, for the cultivars comparison section (left), bacterial and fungal genera that showed significantly higher relative abundance in Frantoio or Picual non-inoculated communities are indicated. For the *V. dahliae* inoculation effect section (right), the most significant changes after pathogen inoculation, at both taxonomic (arrows showing decrease [in red] or increase [in green] in microbial taxa relative abundance for each compartment) and network (arrows showing disappearance [in red] or appearance [in green] in OTUs for each compartment) levels are shown

Differential susceptibility of olive cultivars to *V. dahliae* is mainly attributed to both basal and early pathogen-induced differential transcriptomic responses in the host roots [10], as well as to qualitative and quantitative differential transcriptomic responses of the pathogen when interacting with roots of different cultivars [58]. In this study, a link between VWO tolerance level and the olive belowground-resident microbiome has also been established. On the one hand, our findings stress the need to investigate tolerance to biotic stresses within the holobiont conceptual framework, aiming to a more holistic perspective in tree crop agriculture [59]. On the other hand, results gathered open new perspectives in research lines such as biocontrol and breeding for VWO resistance. As for biocontrol strategies concerns, the fact that the communities associated with the VWO-tolerant cultivar showed a higher abundance of beneficial genera deserves attention as for the identification of novel potential BCA and/or PGPR. Moreover, assessing the effects caused by the introduction of well-characterized BCA against VWO [12–14] on the structure, functionality and network interactions of belowground communities must be investigated as well. This would be relevant for communities exhibiting higher abundance of potential deleterious components as here reported for the VWO-susceptible Picual. Information generated can also be of relevance for olive breeding programs aiming to generate new varieties improved in VWO resistance/tolerance, in which the role of the belowground resident microbiota must be taken into account. Moreover, since propagation of olive plants at the nurseries is mainly and traditionally performed by rooting of stem cuttings, knowledge of the microbial components being recruited to build the root-associated microbiota during the growth of the root system seems of utmost relevance. While the inoculation with *V. dahliae* did not modify the structure (DNA) and the function (RNA) of the olive belowground microbial communities in a noticeable way, microbial co-occurrence interactions showed significant alterations upon pathogen inoculation (a summarizing, simplified scheme is shown in Fig. 5). This work thus shows for the first time a correlation between changes in the root endosphere microbial network topology and the tolerance level of different cultivars to a relevant soilborne pathogen. The detection of *V. dahliae* only in the co-occurrence networks of the VWO-susceptible cultivar communities suggests that the pathogen, when introduced, plays a central role in this community in contrast with Frantoio which manages to confine it out of the most relevant modules. The in-depth study of microbial community co-occurrence interactions has revealed as a powerful tool to unravel the role of the microbiota in tolerance/susceptibility to biotic stress, and we encouraged to be studied in other tree pathosystems.

## Materials and methods

### Sample collection

Olive (3-months old) plants of cultivars Picual and Frantoio, respectively qualified as VWO-susceptible and VWO-tolerant [10] and originating from a commercial nursery located in Córdoba province, were used in the greenhouse experiment. After reception from the nursery, plants were grown in pots (11 × 11 × 12 cm, one plant per pot), each containing a non-sterile, ad hoc prepared soil made of natural soil (70%, w/w) collected at the World Olive Germplasm Collection located at Córdoba municipality [18], sand (7.5%), and a commercial nursery potting substrate (7.5%). Prior to the inoculation with the pathogen, olive plants were acclimated during 3 months in the greenhouse under natural lighting and day/night temperature of 27/21 °C. After this acclimatization period, plants were challenged with isolate *V. dahliae* V-9371, a representative of the D pathotype [60], by adding 150 ml per pot of a conidia suspension (1 × 10^6^ conidial/ml) prepared as previously described [60]. Non-inoculated plants (control) were watered just with 150 ml of water. Root tissues and their associated (rhizosphere) soil of each olive plant were sampled at 0 (four control, non-inoculated plants of each cultivar), and at 8, 15, and 30 (four plants per time-point and per cultivar) days after *V. dahliae inoculation*. Two grams of associated (rhizosphere) soil samples were collected and conserved at − 80 °C in LifeGuard^TM^ Soil Preservation Solution (MoBio Laboratories Inc., Carlsbad, CA, USA) until used. Root samples were collected and washed with 20 ml of NaCl 0.8% by vortex in order to remove the adhering soil. After that, 5 rinses in distilled water were done. Surface sterilization was carried out as follows: 70% alcohol for 5 min, sodium hypochlorite (3.7%), and Tween 20 0.01% for 3 min, and finally 3 rinses in sterile, distilled water. Then, root tissues were immediately frozen in liquid nitrogen and stored at − 80 °C until processing. To confirm the effectiveness of the disinfection protocol, aliquots of the sterile water used in the final rinse were plated onto NA (Nutrient Agar) and LB (Luria Bertani) plates and incubated at 28 °C for 7 days. Then, plates were examined for the presence or absence of contaminating microorganisms.

### DNA and RNA extraction and Illumina sequencing

RNA and DNA from each individual soil sample were obtained using the RNA PowerSoil Total RNA Isolation kit and the RNA PowerSoil DNA Elution accessory kit (MoBio, Laboratories Inc., CA, USA), following the manufacturer’s recommendations within 24 h of samples collection. RNA from each individual root sample was obtained using the RNeasy Plant Mini Kit (Qiagen, Hilden, Germany) and the contaminating genomic DNA was removed by DNase I (Qiagen) treatment followed by a clean-up with the RNeasy MinElute Cleanup kit (Qiagen) according to the manufacturer’s instructions. Pure genomic DNA was extracted using a Nucleon® Phytopure Plant DNA extraction kit (GE Healthcare UK Ltd, Buckinghamshire, UK), following the manufacturer’s recommendations.

cDNA was obtained following the protocol described in Lasa et al. [61]. cDNA and DNA yields and quality were checked both by electrophoresis in 0.8% (w/v) agarose gels stained with GelRed and visualized under UV light, and using a Qubit 3.0 fluorometer (Life Technologies, Grand Island, NY). cDNA and DNA were sequenced using the Illumina MiSeq platform at the genomics service of the Institute of Parasitology and Biomedicine “López Neyra” (CSIC), Granada, Spain. In the first run, prokaryotic libraries were constructed amplifying the hyper-variable regions V3–V4 of the *16S rRNA* gene from both cDNA and DNA using the primer pair Pro341F (5′-CCTACGGGNBGCASCAG-3′) and Pro805R (5′-GACTACNVGGGTATCTAATCC-3′) according to Takahashi et al. [62]. These amplicons were tagged to be attached to PNA PCR clamps to reduce plastid and mitochondrial DNA amplification [63]. In the second run, eukaryotic libraries were constructed amplifying the ITS2 region from both cDNA and DNA using the primer pair ITS4 (5′-TCCTCCGCTTATTGATATGC-3′) [64] and fITS7 (5′-GTGARTCATCGAATCTTTG-3′) [65]. Both runs were sequenced using a paired-end 2 x 300 bp (PE 300) strategy.

### Data quality screening and overlapping

Samples were demultiplexed based on the specific barcode and the Phi-X174-free reads were quality checked with FastQC v.0.11.5 [66] and end-trimmed with FASTX-Toolkit v.0.014 [67]. All low-quality sequences were discarded until reaching a quality value higher than Q20. The paired reads were overlapped with fastq-join v.1.3.1 [68] requesting a minimum overlap of 40 bp and a maximum of 15% of difference in the overlapping region. Both libraries were processed with the same bioinformatics tools but following different pathways detailed below.

### Prokaryotic data processing

Employing the software SEED2 v.2.1.05 [69] and MOTHUR v.1.40.5 [70] the prokaryotic sequences were trimmed and clustered. Using SEED2 the specific primers were discarded and all sequences showing ambiguities, shorter than 384 bp or with an average read quality lower than Q30 were eliminated. After that, with MOTHUR, chimeric reads were removed using SILVA gold reference fasta and the high-quality sequences were clustered into OTU at 97%. Finally, OTU accounting for less than 0.005% of the total sequences were removed according to Bokulich et al. [71]. Furthermore, in the remaining OTU, each OTU that accounted less than 0.005% of sequences in any sample with respect to the total amount of sequences of this OTU were corrected to zero according to the MOCK community used (ZymoBIOMICS Microbial Community Standard II (Log Distribution), ZYMO RESEARCH, CA, USA). OTU were classified with an 80% bootstrap cut off to the Ribosomal Database Project (RDP-II) 16S rRNA reference database, training set v.16 MOTHUR-formatted [72]. Sequences identified as mitochondria, chloroplast, and unknown (unclassified at kingdom level) were removed from the dataset.

### Eukaryotic data processing

The eukaryotic library was quality-trimmed in SEED2 by the removal of sequences with ambiguities and an average read quality lower than Q30. The specific primers and those sequences smaller than 290 bp were eliminated. Subsequently, with the tool VSEARCH “De Novo” implemented in MOTHUR, chimeric sequences were identified and discarded and the good quality sequences were distance-based greedy clustered at 97% similarity. The most abundant OTU sequences were classified using the UNITE v.7.2 dynamic database [73] following the parameters recommended in the website and used by Findley et al. [74]. The same OTU trimming than in prokaryotic data was applied in the OTU table. Finally, only OTU assigned to kingdom Fungi were conserved for further analyses.

### Core microbiome construction

The DNA and RNA core bacteriome and mycobiome were built considering only genera that were present in 90% of the replicates of each treatment at minimum [75]. The shared core genera were present in both cultivars and the specific ones were present in one cultivar but missing in more than 10% of the replicates of the other cultivar. After construction, core microbiomes were plotted in Venn diagrams.

### Statistical analyses

All analyses were performed with scripts previously described by Fernández-González et al*.* [18]. Briefly, alpha diversity indices (Observed and Chao1 richness; Shannon and InvSimpson) were compared with Kruskal-Wallis test and *p* values were FDR corrected by the Benjamini-Hochberg method using the R package *agricolae*. For the beta diversity, a normalization of the filtered OTU sequence counts was performed using the “trimmed means of M” (TMM) method with the BioConductor package *edgeR*. The normalized data were considered to perform the permutational analysis of variance (PERMANOVA) and permutational analysis of multivariate homogeneity of groups dispersions (BETADISPER) using the functions *adonis* and *betadisper* in the vegan package with 9999 permutations. Where applicable, pairwise differences between groups were assessed with the function *pairwise adonis* from the package *pairwiseAdonis*. To visualize the similarities or dissimilarities of the studied communities, those which resulted significant from the PERMANOVA analyses were plotted by Non-metric MultiDimensional Scaling Analysis (NMDS) and Principal Coordinates Analysis (PCoA). Bray-Curtis dissimilarities were used to ordinate in two dimensions the variance of beta diversity among all treatments. Ordination analyses were performed using the R package *phyloseq*. For each significant PERMANOVA comparison, NMDS or PCoA was chosen depending on which one best represented the results of the permutational analysis. Biologically relevant prokaryotic or fungal phyla, orders, and genera were obtained testing for differential taxa abundance using proportions in non-normalized counts with the STAMP v.2.1.3 software, selecting ANOVA Games-Howell’s post hoc test parameters for multiple groups and Welch’s *t* test for two groups comparisons, considering Benjamini-Hochberg FDR for multiple tests correction. Taxa with statistically significant differences in the two methods previously described were filtered to keep only those ones in which the difference between proportions was ≥ 0.5%, or the ratio of proportions was ≥ 2 to be considered biologically relevant and to generate the final selection.

### Network construction, comparison, and visualization

First of all, bacterial and fungal networks were separately constructed for each cultivar (Picual and Frantoio), each compartment (root endosphere and rhizosphere), each nucleic acid (DNA and RNA), and each treatment (control and *V. dahliae*-inoculated). In every network, all time-points and replicates (*n* = 16 in control plants and *n* = 12 in inoculated plants), excluding Fra_3_0_P in Frantoio DNA control (*n* = 15), Pic_1_30_S in Picual DNA and RNA inoculated (*n* = 11), were considered to obtain a more accurate correlation between different OTUs. Then, to build these 16 networks, MENAP website was used (http://ieg4.rccc.ou.edu/mena/main.cgi) following the developer’s recommendations [28, 31, 76–78]. The only parameter changed from default options was the separation method. Indeed, simulated annealing approach was selected instead of greedy modularity optimization as recommended by Jiemeng et al. [28]. Moreover, 100 random networks were performed to each empirical network to use the standard deviation of the global properties in Student *t* test comparisons of the empirical networks between cultivars. All the networks were drawn by using Cytoscape v.3.7.1 [78]. Finally, keystone OTU were plotted in Excel (ZiPi plots) and compared between treatments in each cultivar, each compartment and nucleic acid.

## Supplementary information

**Additional file 1: Table S1**. Codes and description of the studied samples. The same codes were used for bacterial and fungal communities.

**Additional file 2: Figure S1**. Phyla showing significant changes in the bacterial structural (DNA) community of the ‘Picual’ rhizosphere after inoculation with *Verticillium dahliae*. No/green: non-inoculated; Yes/red: *Verticillium dahliae*-inoculated.

**Additional file 3: Table S2**. Core bacteriome of 'Frantoio' (yellow cells) and 'Picual' (green cells) cultivars in non-inoculated conditions. The shared core bacteriome is shown with light brown cells. Genera in red color belong to the RNA (functional community) core. In bold type letters main genera with relative abundance > 1% (sum of both cultivars).

**Additional file 4: Table S3**. Core bacteriome of 'Frantoio' (yellow cells) and 'Picual' (green cells) cultivars upon *Verticillium dahliae* inoculation. The shared core bacteriome is shown with light brown cells. Genera in red color belong to the RNA (functional community) core. In bold type letters main genera with relative abundance > 1% (sum of both cultivars).

**Additional file 5: Figure S2**. Genera showing significant differences between non-inoculated 'Frantoio' (green) and 'Picual' (blue) plants. The root endosphere structural (panel a) and functional (panel b) bacterial communities are shown.

**Additional file 6: Figure S3**. Genera showing significant changes in bacterial structural (DNA) community of ‘Frantoio’ (panel a) and ‘Picual’ (panel b) endosphere after inoculation with *Verticillium dahliae*. No/green: non-inoculated; Yes/red: *Verticillium dahliae*-inoculated.

**Additional file 7: Figure S4**. Genera showing significant differences between non-inoculated 'Frantoio' (green) and 'Picual' (blue) plants. The root rhizosphere structural (panel a) and functional (panel b) bacterial communities are shown.

**Additional file 8: Figure S5**. Genera with significantly different changes in rhizosphere of Frantoio and Picual bacterial structural (panels a and c respectively) and functional (panels b and d respectively) communities after inoculation with *Verticillium dahliae*. No/green: non-inoculated; Yes/red: *Verticillium dahliae*-inoculated.

**Additional file 9: Table S4**. Core mycobiome of 'Frantoio' (yellow cells) and 'Picual' (green cells) cultivars in non-inoculated conditions. The shared core bacteriome is shown with light brown cells. Genera in red color belong to the RNA (functional community) core. In bold type letters main genera with relative abundance > 1% (sum of both cultivars).

**Additional file 10: Table S5**. Core mycobiome of 'Frantoio' (yellow cells) and 'Picual' (green cells) cultivars upon *Verticillium dahliae* inoculation. The shared core bacteriome is shown with light brown cells. Genera in red color belong to the RNA (functional community) core. In bold type letters main genera with relative abundance > 1% (sum of both cultivars).

**Additional file 11: Figure S6**. Genera showing significant differences between non-inoculated 'Frantoio' (bordeaux) and 'Picual' (violet) plants. The root endosphere structural (panel a) and functional (panel b) fungal communities are shown.

**Additional file 12: Figure S7**. Genera showing significant differences between non-inoculated 'Frantoio' (bordeaux) and 'Picual' (violet) plants. The root rhizosphere structural (panel a) and functional (panel b) bacterial communities are shown.

**Additional file 13: Figure S8**. Genera showing significant changes in fungal structural (DNA) and functional (RNA) communities of ‘Frantoio’ (panel a) and ‘Picual’ (panel b) rhizosphere after inoculation with *Verticillium dahliae*. No/green: non-inoculated; Yes/red: *Verticillium dahliae*-inoculated.

**Additional file 14: Figure S9**. ZiPi plots highlighting the keystone OTUs of the root endosphere microbial structural (a) and functional (b) communities from Frantoio upon inoculation with *Verticillium dahliae*. In the table are the details of each keystone OTU.

**Additional file 15: Figure S10**. ZiPi plots highlighting the keystone OTUs of the root endosphere microbial structural (a) and functional (b) communities from Picual upon inoculation with *Verticillium dahliae*. In the table are the details of each keystone OTU.

**Additional file 16: Figure S11**. Co-occurrence networks of functional (RNA) communities from root endosphere of both cultivars before and after inoculation.

**Additional file 17: Figure S12**. Co-occurrence networks of functional (RNA) communities from rhizosphere of both cultivars before and after inoculation.

## Data Availability

The datasets generated and analyzed during the current study are available in the NCBI Sequence Read Archive (SRA) under the BioProject number PRJNA543961.
