# Supplementary material for: Linking belowground microbial network changes to different tolerance level towards Verticillium wilt of olive
Source: Microbiome. 2020 Feb 1;8:11. doi: 10.1186/s40168-020-0787-2 (PMC6995654; doi:10.1186/s40168-020-0787-2)
Supplement: Supplementary file 6 — Additional file 5: Figure S2. Genera showing significant differences between non-inoculated 'Frantoio' (green) and 'Picual' (blue) plants. The root endosphere structural (panel a) and functional (panel b) bacterial communities are shown. [file 40168_2020_787_MOESM5_ESM.pdf]

Figure S2. Genera showing significant differences between non-inoculated 'Frantoio' (green) and 'Picual' (blue) plants. The root endosphere structural (panel a) and functional (panel b) bacterial communities are shown.

a

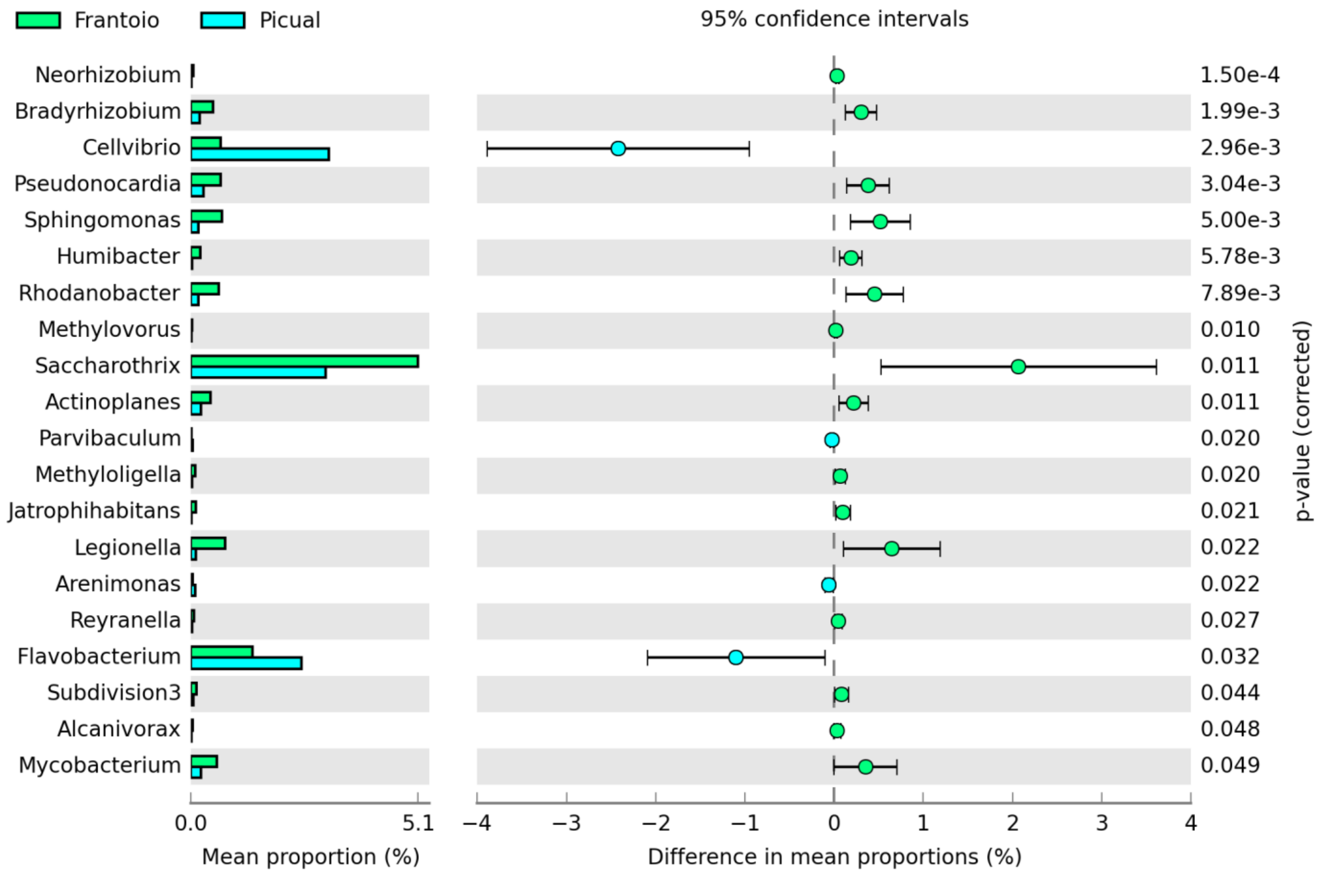

b

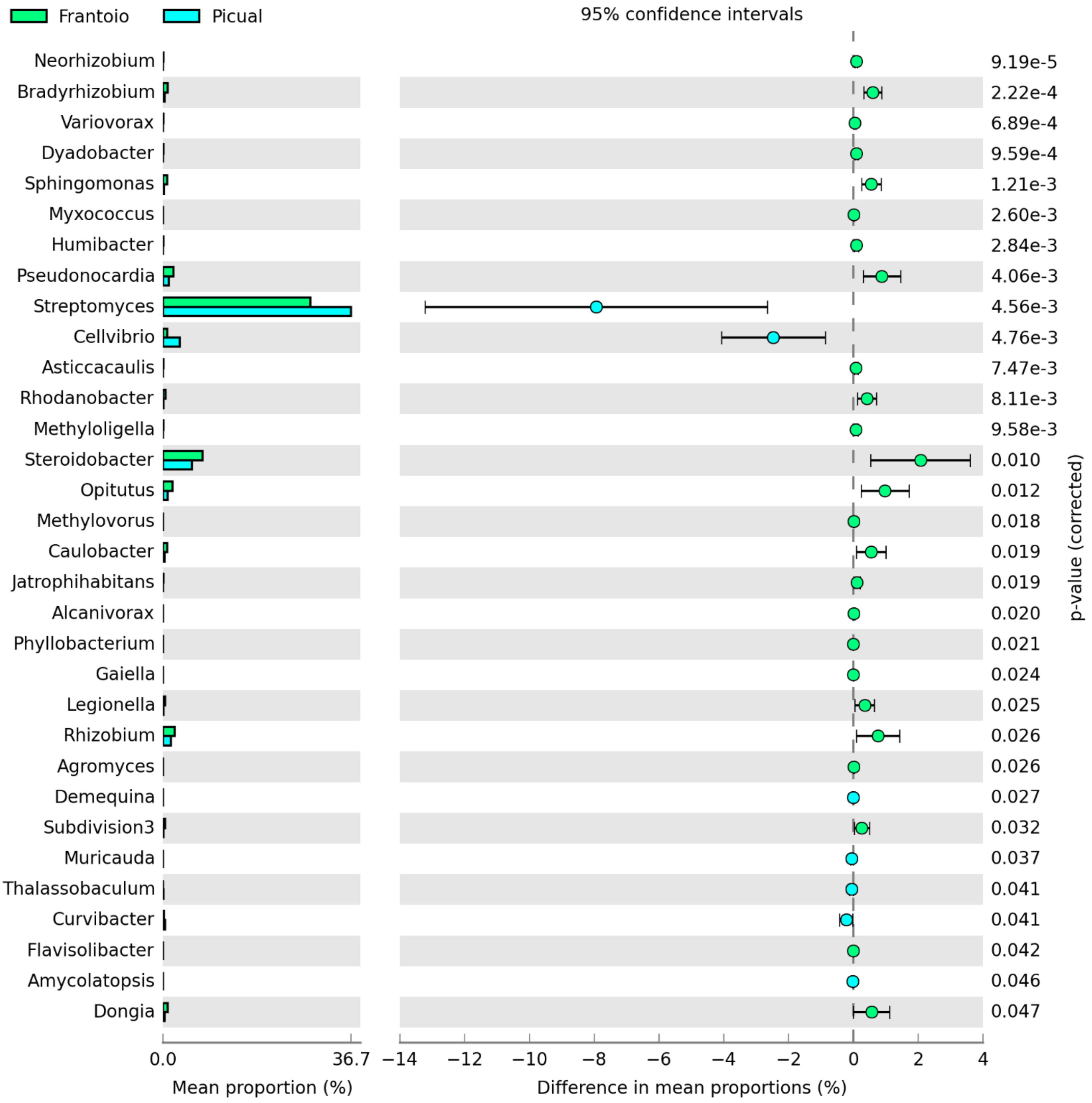
Frantoio
Picual
95% confidence intervals
Neorhizobium 9.19e-5
Bradyrhizobium 2.22e-4
Variovorax 6.89e-4
Dyadobacter 9.59e-4
Sphingomonas 1.21e-3
Myxococcus 2.60e-3
Humibacter 2.84e-3
Pseudonocardia 4.06e-3
Streptomyces 4.56e-3
Cellvibrio 4.76e-3
Asticcacaulis 7.47e-3
Rhodanobacter 8.11e-3
Methyloligella 9.58e-3
Steroidobacter 0.010
Opitutus 0.012
Methylovorus 0.018
Caulobacter 0.019
Jatrophihabitans 0.019
Alcanivorax 0.020
Phyllobacterium 0.021
Gaiella 0.024
Legionella 0.025
Rhizobium 0.026
Agromyces 0.026
Demequina 0.027
Subdivision3 0.032
Muricauda 0.037
Thalassobaculum 0.041
Curvibacter 0.041
Flavisolibacter 0.042
Amycolatopsis 0.046
Dongia 0.047
p-value (corrected)
0.0
36.7
Mean proportion (%)
−14 −12 −10 −8 −6 −4 −2 0 2 4
Difference in mean proportions (%)
