# Supplementary material for: Linking belowground microbial network changes to different tolerance level towards Verticillium wilt of olive
Source: Microbiome. 2020 Feb 1;8:11. doi: 10.1186/s40168-020-0787-2 (PMC6995654; doi:10.1186/s40168-020-0787-2)

Figure S3. Genera showing significant changes in bacterial structural (DNA) community of ‘Frantoio’ (panel a) and ‘Picual’ (panel b) endosphere after inoculation with *Verticillium dahliae*. No/green: non-inoculated; Yes/red: *Verticillium dahliae*-inoculated.

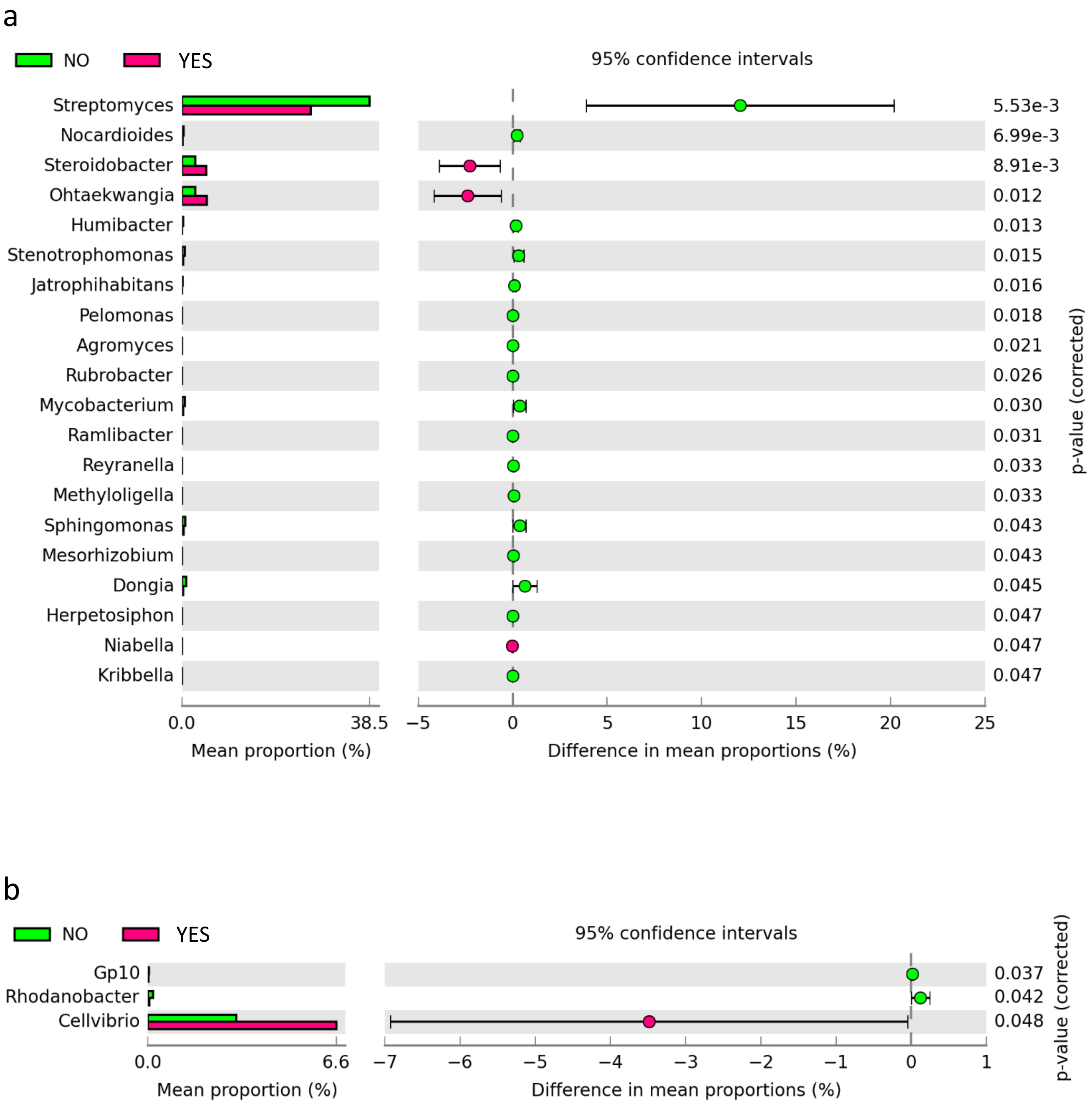

Supplement: Supplementary file 7 — Additional file 6: Figure S3. Genera showing significant changes in bacterial structural (DNA) community of ‘Frantoio’ (panel a) and ‘Picual’ (panel b) endosphere after inoculation with Verticillium dahliae. No/green: non-inoculated; Yes/red: Verticillium dahliae-inoculated. [file 40168_2020_787_MOESM6_ESM.pdf]
