# Supplementary material for: Linking belowground microbial network changes to different tolerance level towards Verticillium wilt of olive
Source: Microbiome. 2020 Feb 1;8:11. doi: 10.1186/s40168-020-0787-2 (PMC6995654; doi:10.1186/s40168-020-0787-2)

Figure S5. Genera with significantly different changes in rhizosphere of Frantoio and Picual bacterial structural (panels a and c respectively) and functional (panels b and d respectively) communities after inoculation with *Verticillium dahliae*. No/green: non-inoculated; Yes/red: *Verticillium dahliae*-inoculated.

a

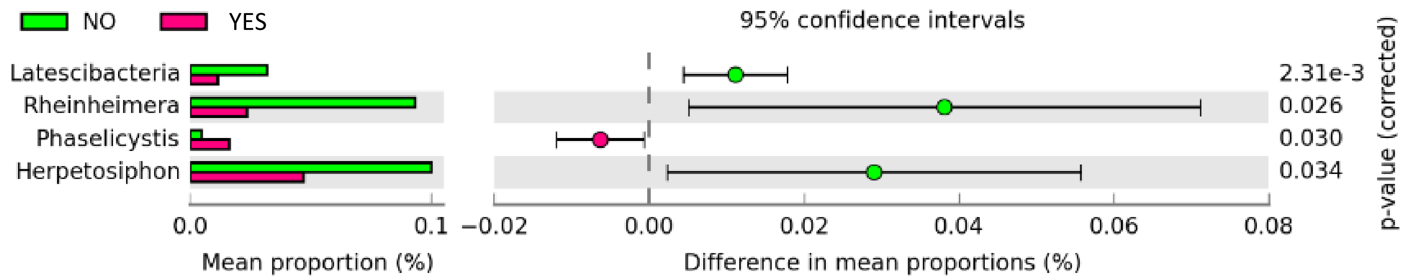

b

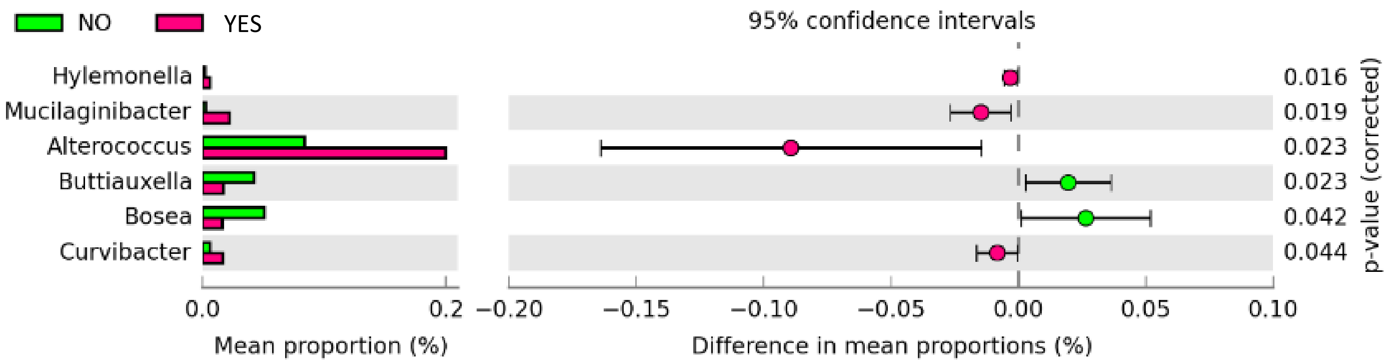

c

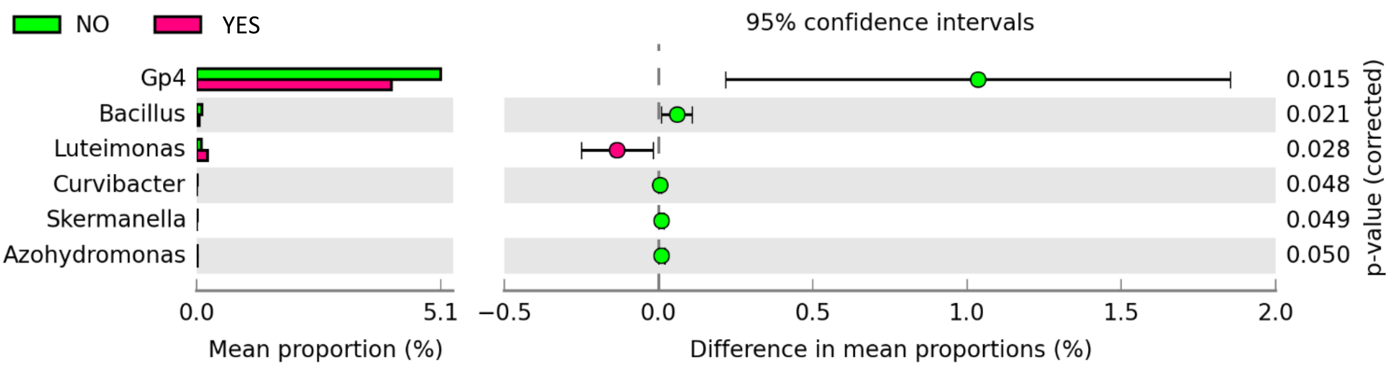

d

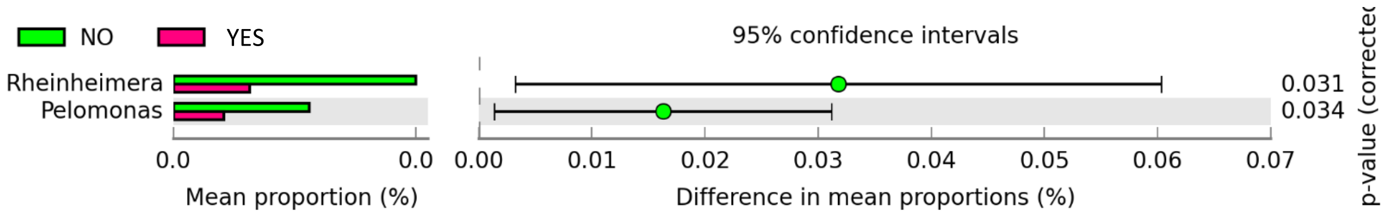

Supplement: Supplementary file 9 — Additional file 8: Figure S5. Genera with significantly different changes in rhizosphere of Frantoio and Picual bacterial structural (panels a and c respectively) and functional (panels b and d respectively) communities after inoculation with Verticillium dahliae. No/green: non-inoculated; Yes/red: Verticillium dahliae-inoculated. [file 40168_2020_787_MOESM8_ESM.pdf]
