# Supplementary material for: Linking belowground microbial network changes to different tolerance level towards Verticillium wilt of olive
Source: Microbiome. 2020 Feb 1;8:11. doi: 10.1186/s40168-020-0787-2 (PMC6995654; doi:10.1186/s40168-020-0787-2)

Figure S7. Genera showing significant differences between non-inoculated 'Frantoio' (bordeaux) and 'Picual' (violet) plants. The root rhizosphere structural (panel a) and functional (panel b) bacterial communities are shown.

a

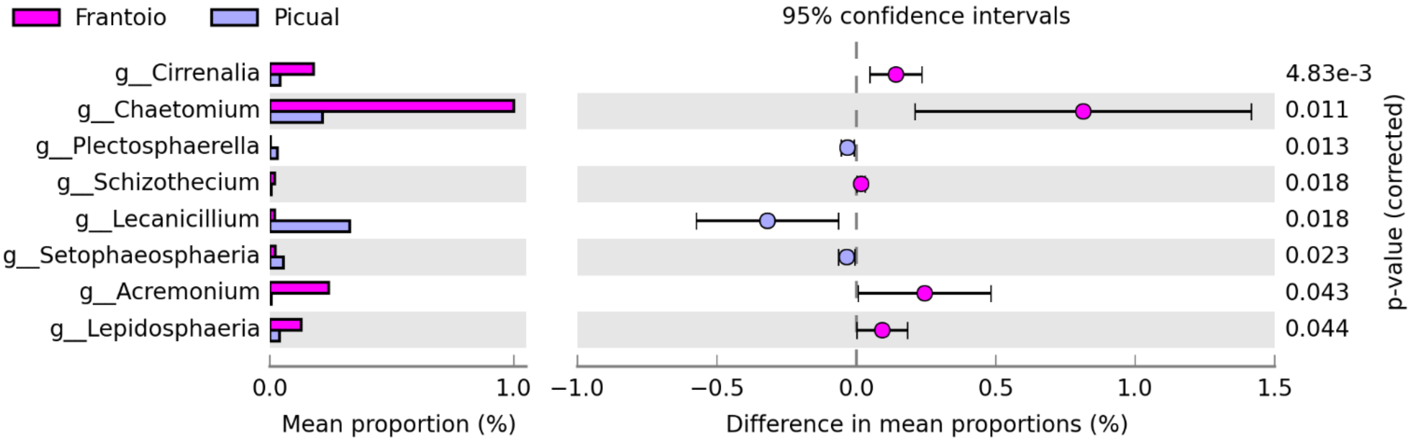

b

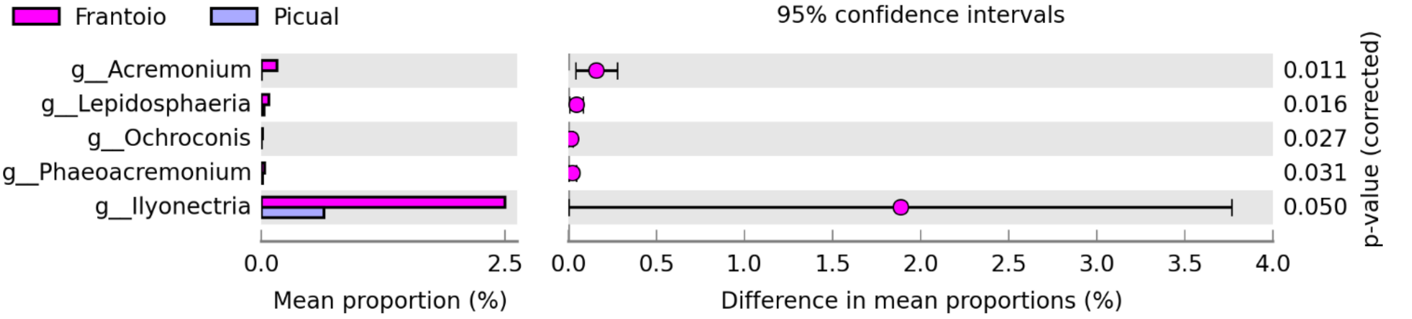

Supplement: Supplementary file 13 — Additional file 12: Figure S7. Genera showing significant differences between non-inoculated 'Frantoio' (bordeaux) and 'Picual' (violet) plants. The root rhizosphere structural (panel a) and functional (panel b) bacterial communities are shown. [file 40168_2020_787_MOESM12_ESM.pdf]
