# Supplementary material for: Linking belowground microbial network changes to different tolerance level towards Verticillium wilt of olive
Source: Microbiome. 2020 Feb 1;8:11. doi: 10.1186/s40168-020-0787-2 (PMC6995654; doi:10.1186/s40168-020-0787-2)
Supplement: Supplementary file 14 — Additional file 13: Figure S8. Genera showing significant changes in fungal structural (DNA) and functional (RNA) communities of ‘Frantoio’ (panel a) and ‘Picual’ (panel b) rhizosphere after inoculation with Verticillium dahliae. No/green: non-inoculated; Yes/red: Verticillium dahliae-inoculated. [file 40168_2020_787_MOESM13_ESM.pdf]

Figure S8. Genera showing significant changes in fungal structural (DNA) and functional (RNA) communities of 'Frantoio' (panel a) and 'Picual' (panel b) rhizosphere after inoculation with *Verticillium dahliae*. No/green: non-inoculated; Yes/red: *Verticillium dahliae*-inoculated.

a

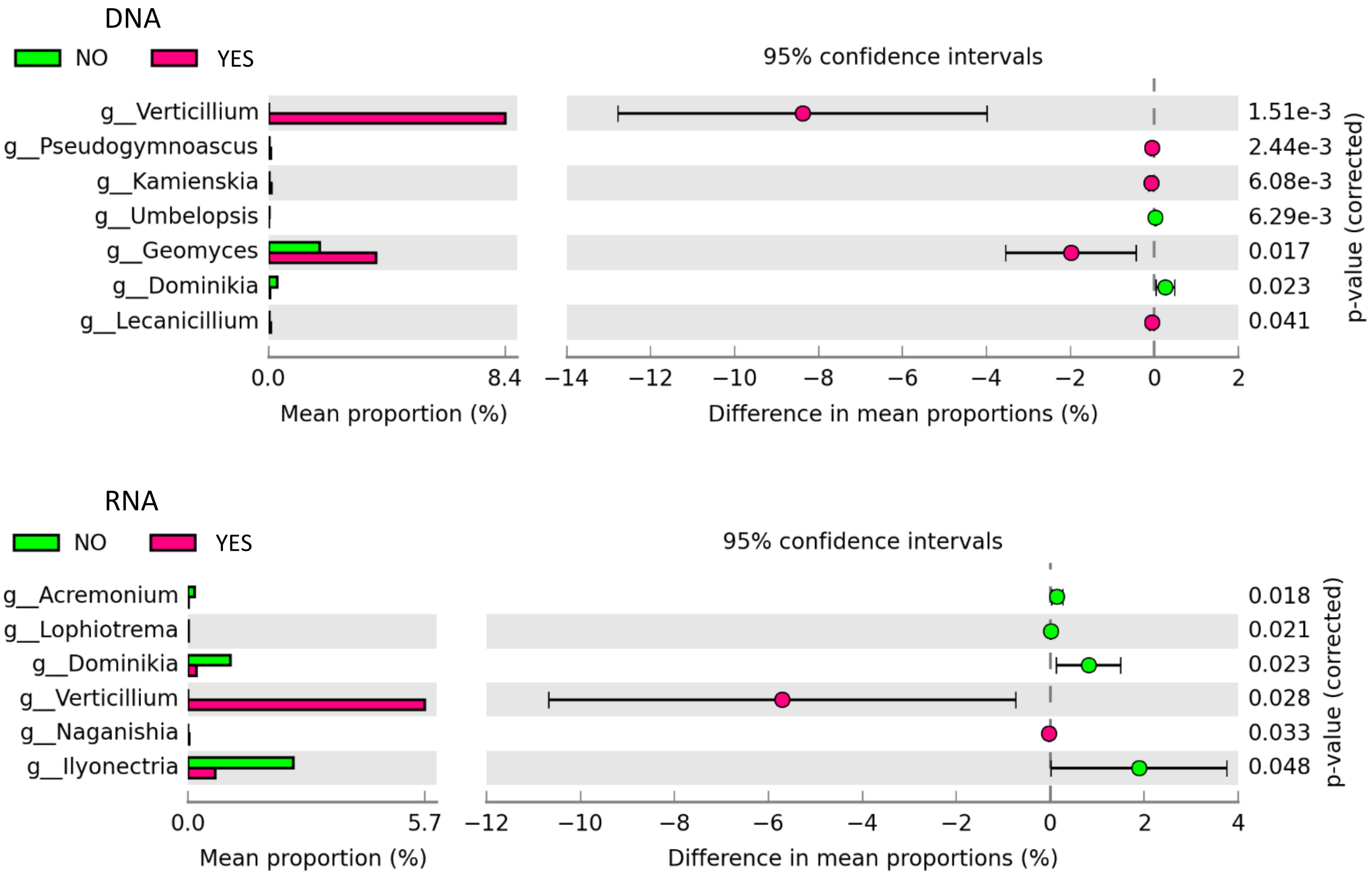

b

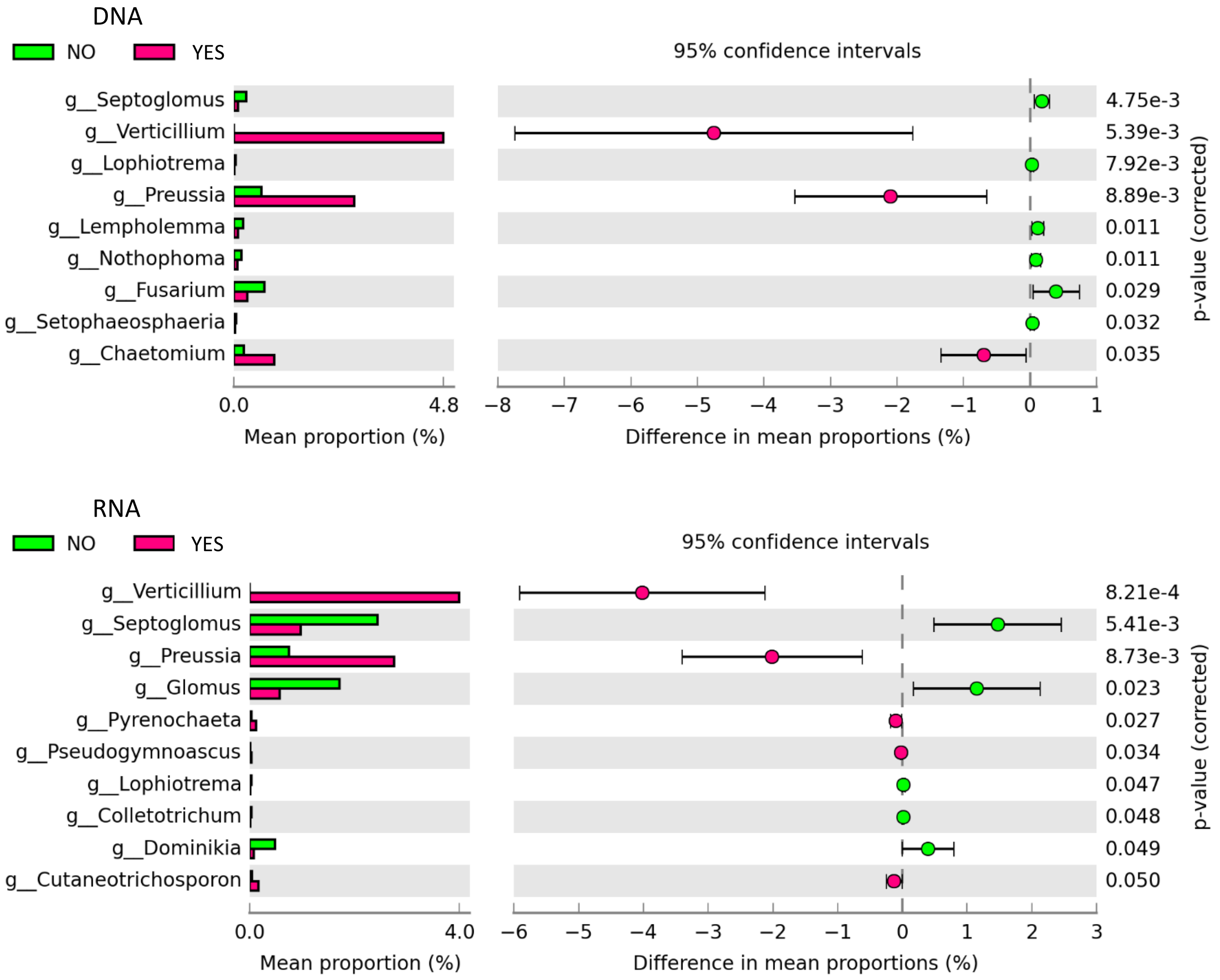
DNA
NO
YES
95% confidence intervals
g__Septoglomus
g__Verticillium
g__Lophiotrema
g__Preussia
g__Lempholemma
g__Nothophoma
g__Fusarium
g__Setophaeosphaeria
g__Chaetomium
4.75e-3
5.39e-3
7.92e-3
8.89e-3
0.011
0.011
0.029
0.032
0.035
p-value (corrected)
0.0
4.8
Mean proportion (%)
−8
−7
−6
−5
−4
−3
−2
−1
0
1
Difference in mean proportions (%)
RNA
NO
YES
95% confidence intervals
g__Verticillium
g__Septoglomus
g__Preussia
g__Glomus
g__Pyrenochaeta
g__Pseudogymnoascus
g__Lophiotrema
g__Colletotrichum
g__Dominikia
g__Cutaneotrichosporon
8.21e-4
5.41e-3
8.73e-3
0.023
0.027
0.034
0.047
0.048
0.049
0.050
p-value (corrected)
0.0
4.0
Mean proportion (%)
−6
−5
−4
−3
−2
−1
0
1
2
3
Difference in mean proportions (%)
