# Supplementary material for: Linking belowground microbial network changes to different tolerance level towards Verticillium wilt of olive
Source: Microbiome. 2020 Feb 1;8:11. doi: 10.1186/s40168-020-0787-2 (PMC6995654; doi:10.1186/s40168-020-0787-2)
Supplement: Supplementary file 15 — Additional file 14: Figure S9. ZiPi plots highlighting the keystone OTUs of the root endosphere microbial structural (a) and functional (b) communities from Frantoio upon inoculation with Verticillium dahliae. In the table are the details of each keystone OTU. [file 40168_2020_787_MOESM14_ESM.pdf]

Figure S9. ZiPi plots highlighting the keystone OTUs of the root endosphere microbial structural (a) and functional (b) communities from Frantoio upon inoculation with *Verticillium dahliae*. In the tables are the details of each keystone OTU.

a

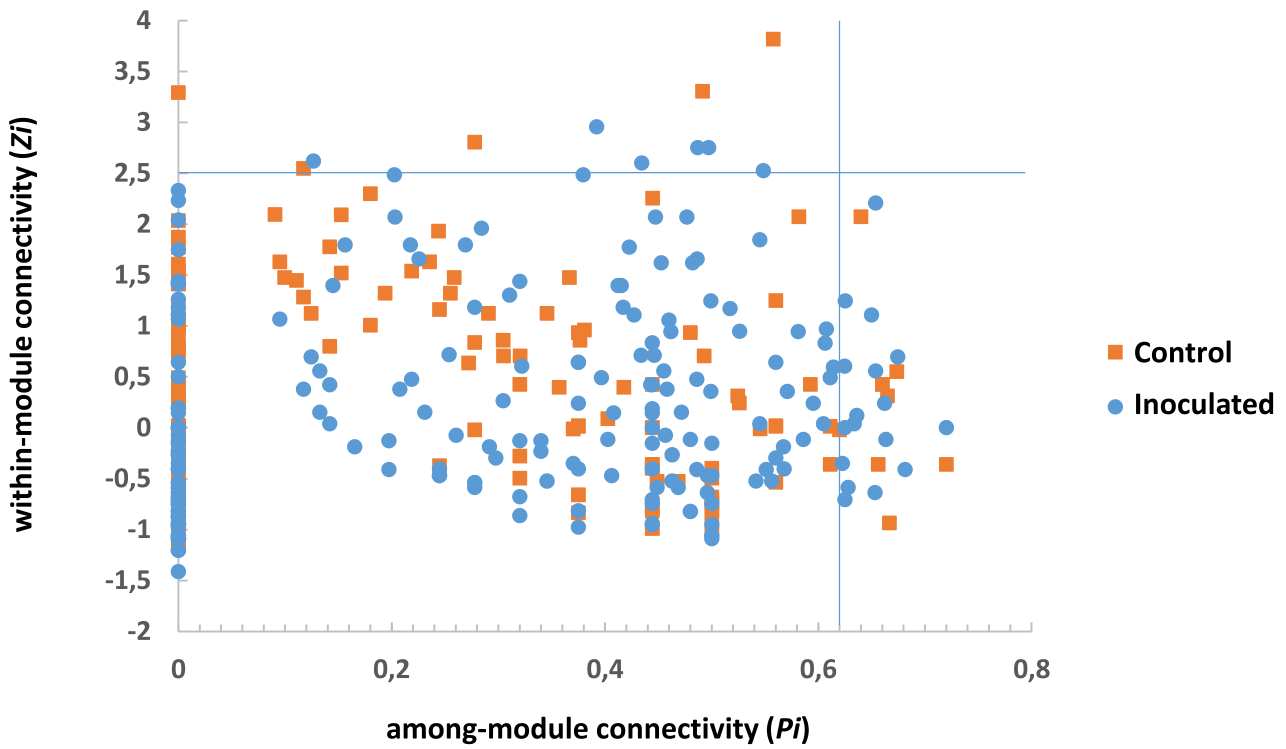

	Name	Phylum	Genus	No. module
Module hubs	b_Otu000046	Actinobacteria	unclassified	3
	b_Otu000058	Proteobacteria	Phenylobacterium	4
	f_Otu00080	Glomeromycota	Glomus	6
	b_Otu000132	Proteobacteria	Xanthomonas	5
	f_Otu00002	Glomeromycota	Rhizophagus	2
Connectors	b_Otu000093	Proteobacteria	Ferrovibrio	4
	b_Otu000031	Proteobacteria	Devosia	1
	b_Otu000119	Verrucomicrobia	Roseimicrobium	4
	b_Otu000036	Proteobacteria	Sphingomonas	0
	b_Otu000323	Proteobacteria	unclassified	2
	b_Otu000076	Proteobacteria	Devosia	3
	b_Otu000042	Proteobacteria	unclassified	5
	b_Otu000051	Proteobacteria	Devosia	4
	b_Otu000127	Proteobacteria	Hydrogenophaga	3
Module hubs	b_Otu000117	Proteobacteria	Steroidobacter	2
	b_Otu000062	unclassified	unclassified	1
	b_Otu000001	Actinobacteria	Streptomyces	1
	b_Otu000992	Verrucomicrobia	Opitutus	0
	b_Otu000005	Proteobacteria	Steroidobacter	2
	b_Otu000008	unclassified	unclassified	1
Connectors	b_Otu001222	Actinobacteria	unclassified	0
	b_Otu000044	Proteobacteria	Rhizobium	0
	b_Otu000540	Verrucomicrobia	Prosthecobacter	0
	b_Otu000970	Verrucomicrobia	Opitutus	1
	b_Otu000020	Proteobacteria	Pseudomonas	0
	b_Otu000206	Bacteroidetes	unclassified	2
	b_Otu000606	Proteobacteria	unclassified	1
	b_Otu000779	Proteobacteria	Brevundimonas	15
	b_Otu001354	Proteobacteria	Ancylobacter	15
	b_Otu000433	Proteobacteria	unclassified	2
	b_Otu000121	Proteobacteria	Legionella	2
	b_Otu000677	Proteobacteria	unclassified	1
	b_Otu000490	Bacteroidetes	Dyadobacter	2
	b_Otu000067	Proteobacteria	Hylemonella	1
	b_Otu000360	Bacteroidetes	unclassified	16
	b_Otu000051	Proteobacteria	Devosia	0
	b_Otu000127	Proteobacteria	Hydrogenophaga	2

b

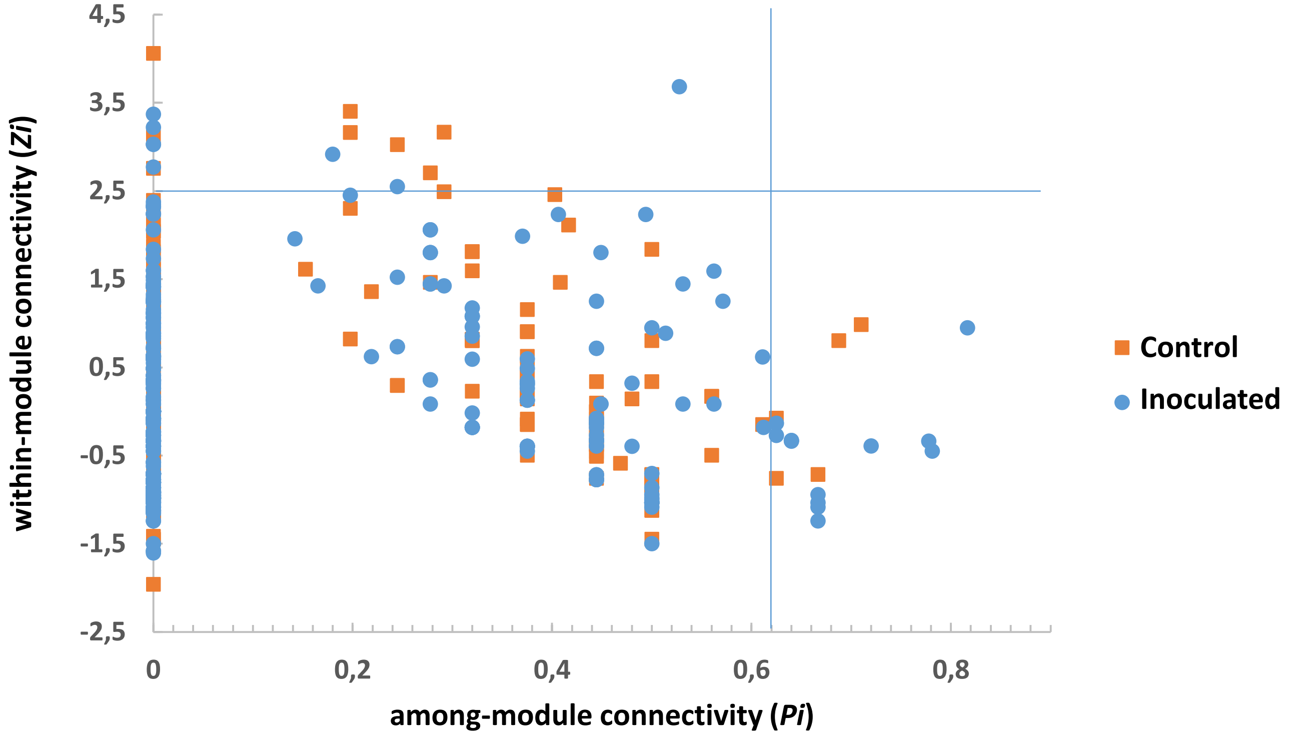

	Name	Phylum	Genus	No. module
Module hubs	b_Otu000135	Bacteroidetes	unclassified	2
	b_Otu000093	Proteobacteria	Ferrovibrio	1
	b_Otu000067	Proteobacteria	Hylemonella	6
	b_Otu000817	Proteobacteria	unclassified	9
	b_Otu000974	Proteobacteria	Luteimonas	11
	b_Otu000117	Proteobacteria	Steroidobacter	10
	b_Otu000688	Bacteroidetes	Terrimonas	8
	b_Otu000473	unclassified	unclassified	4
Connectors	b_Otu000911	unclassified	unclassified	0
	b_Otu000059	Proteobacteria	Steroidobacter	4
	f_Otu00081	Ascomycota	unclassified_Hypocreales	9
	b_Otu000695	Actinobacteria	unclassified	9
	b_Otu001227	Proteobacteria	Myxococcus	3
Module hubs	b_Otu000533	Proteobacteria	Methylophilus	4
	b_Otu001084	Verrucomicrobia	unclassified	1
	f_Otu00478	Ascomycota	unclassified_Pleosporales	2
	b_Otu000045	Actinobacteria	Pseudonocardia	0
	b_Otu000063	Proteobacteria	Steroidobacter	0
	b_Otu000344	Bacteroidetes	Flavobacterium	3
	b_Otu000183	Proteobacteria	unclassified	5
	b_Otu000088	Actinobacteria	Solirubrobacter	12
Connectors	f_Otu00142	Ascomycota	Cirrenalia	6
	b_Otu000082	unclassified	unclassified	8
	f_Otu00001	Ascomycota	unclassified_Pleosporales	2
	f_Otu00264	Basidiomycota	unclassified_Ceratobasidiaceae	2
	b_Otu000043	Proteobacteria	Bradyrhizobium	3
	b_Otu000428	Proteobacteria	unclassified	3
	b_Otu000598	Bacteroidetes	Pedobacter	12
	f_Otu00075	Basidiomycota	unclassified_Sebacinales	0
	b_Otu000517	Proteobacteria	unclassified	10
	b_Otu000700	Verrucomicrobia	Subdivision3	5
	f_Otu00126	Ascomycota	Conlarium	7
	b_Otu000259	Actinobacteria	Nocardioides	13
	b_Otu000473	unclassified	unclassified	5
