# Supplementary material for: Linking belowground microbial network changes to different tolerance level towards Verticillium wilt of olive
Source: Microbiome. 2020 Feb 1;8:11. doi: 10.1186/s40168-020-0787-2 (PMC6995654; doi:10.1186/s40168-020-0787-2)
Supplement: Supplementary file 16 — Additional file 15: Figure S10. ZiPi plots highlighting the keystone OTUs of the root endosphere microbial structural (a) and functional (b) communities from Picual upon inoculation with Verticillium dahliae. In the table are the details of each keystone OTU. [file 40168_2020_787_MOESM15_ESM.pdf]

Figure S10. ZiPi plots highlighting the keystone OTUs of the root endosphere microbial structural (a and c) and functional (b and d) communities from Picual upon inoculation with *Verticillium dahliae*. In the tables are the details of each keystone OTU.

a

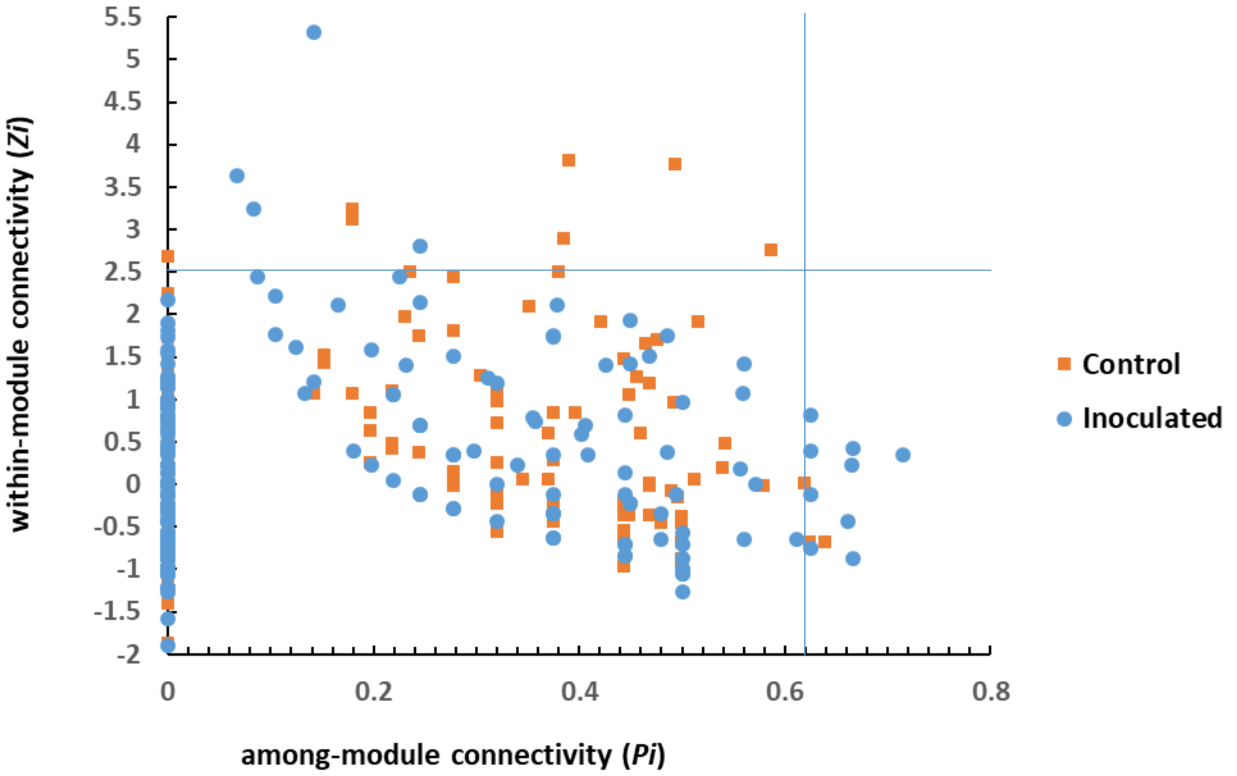

	Name	Phylum	Genus	No. module
Module hubs	b_Otu000032	Proteobacteria	unclassified	0
	b_Otu000022	Proteobacteria	Stenotrophomonas	3
	f_Otu00284	Glomeromycota	Rhizophagus	6
	b_Otu000119	Verrucomicrobia	Roseimicrobium	0
	b_Otu000076	Proteobacteria	Devosia	0
	b_Otu000005	Proteobacteria	Steroidobacter	1
	f_Otu00080	Glomeromycota	Glomus	5
Connectors	b_Otu000659	Proteobacteria	Luteimonas	3
	b_Otu000256	Actinobacteria	Actinoplanes	3
	b_Otu000392	Firmicutes	Bacillus	3
Module hubs	f_Otu00032	Ascomycota	unclassified	2
	b_Otu000031	Proteobacteria	Devosia	0
	b_Otu001155	unclassified	unclassified	1
	b_Otu000102	Proteobacteria	unclassified	3
Connectors	b_Otu000376	Proteobacteria	unclassified	11
	b_Otu000051	Proteobacteria	Devosia	2
	b_Otu000245	Proteobacteria	unclassified	0
	b_Otu001307	Proteobacteria	Peredibacter	3
	b_Otu000054	Proteobacteria	unclassified	0
	b_Otu000047	Proteobacteria	Rhodanobacter	2
	b_Otu000201	Bacteroidetes	Flavobacterium	1
	b_Otu000323	Proteobacteria	unclassified	12
	b_Otu000714	Proteobacteria	Sphingomonas	4

b

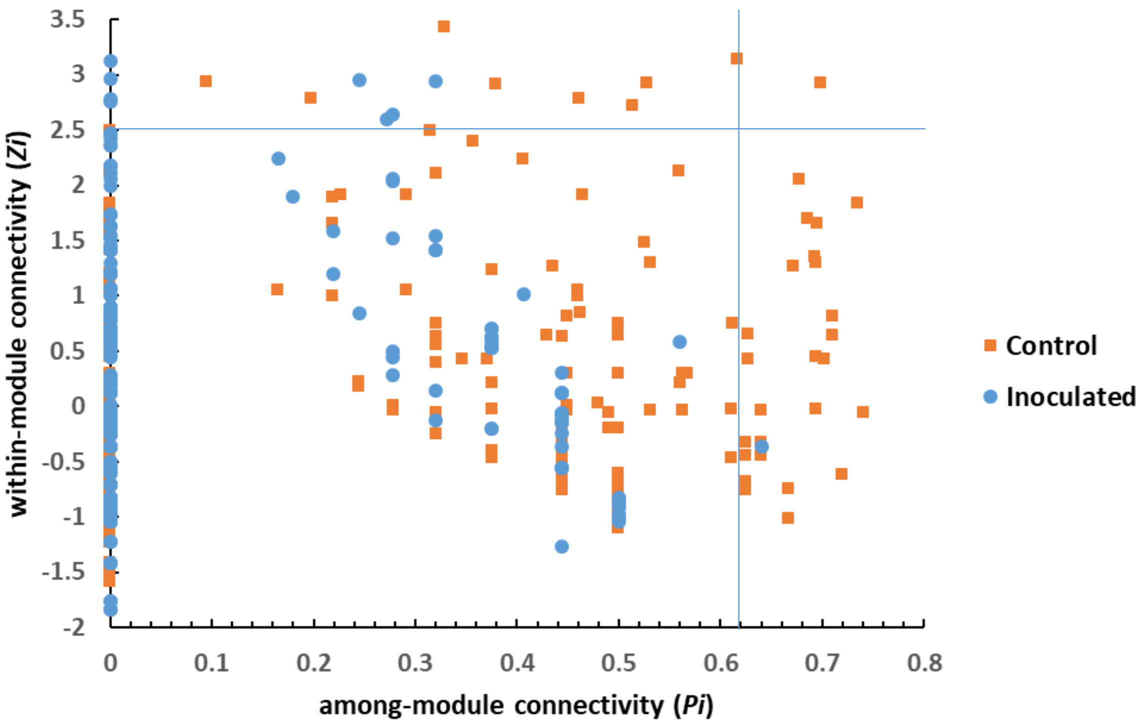

	Name	Phylum	Genus	No. module
Network hub	b_Otu000055	*Verrucomicrobia*	*Opitutus*	2
Module hubs	b_Otu000031	*Proteobacteria*	*Devosia*	1
	b_Otu000045	*Actinobacteria*	*Pseudonocardia*	5
	b_Otu000049	*Proteobacteria*	*Caulobacter*	0
	b_Otu000111	*Proteobacteria*	*Methylophilus*	3
	b_Otu000147	*unclassified*	*unclassified*	1
	b_Otu000202	*Actinobacteria*	*Mycobacterium*	0
	b_Otu000274	*Proteobacteria*	*unclassified*	2
	b_Otu000280	*Verrucomicrobia*	*Subdivision3*	1
Connectors	b_Otu000032	*Proteobacteria*	*unclassified*	4
	b_Otu000076	*Proteobacteria*	*Devosia*	3
	b_Otu000081	*Actinobacteria*	*Pseudonocardia*	5
	b_Otu000082	*unclassified*	*unclassified*	1
	b_Otu000088	*Actinobacteria*	*Solirubrobacter*	3
	b_Otu000108	*Verrucomicrobia*	*Opitutus*	4
	b_Otu000120	*Proteobacteria*	*unclassified*	4
	b_Otu000123	*Proteobacteria*	*Hyphomicrobium*	4
	b_Otu000181	*Proteobacteria*	*unclassified*	0
	b_Otu000183	*Proteobacteria*	*unclassified*	0
	b_Otu000204	*Proteobacteria*	*unclassified*	1
	b_Otu000225	*Bacteroidetes*	*Flavobacterium*	3
	b_Otu000389	*Actinobacteria*	*unclassified*	6
	b_Otu000414	*Actinobacteria*	*unclassified*	1
	b_Otu000427	*Proteobacteria*	*unclassified*	0
	b_Otu000460	*Bacteroidetes*	*unclassified*	2
	b_Otu000533	*Proteobacteria*	*Methylophilus*	3
	b_Otu000705	*Acidobacteria*	*Gp6*	0
	b_Otu000732	*Proteobacteria*	*unclassified*	2
	b_Otu000878	*Firmicutes*	*Bacillus*	2
	b_Otu000960	*Bacteroidetes*	*unclassified*	1
	b_Otu001046	*Actinobacteria*	*unclassified*	2
	b_Otu001057	*Acidobacteria*	*Gp10*	6
	b_Otu001114	*Bacteroidetes*	*Flavobacterium*	7
	b_Otu001158	*Verrucomicrobia*	*Prosthecobacter*	4
	**f_Otu00260**	***Ascomycota***	***unclassified_Hypocreales***	**4**
Module hubs	b_Otu000046	*Actinobacteria*	*Microbacteriaceae*	0
	b_Otu000096	*Proteobacteria*	*Asticcacaulis*	6
	b_Otu000212	*Proteobacteria*	*Hyphomicrobiaceae*	9
	b_Otu000252	*Proteobacteria*	*Thalassobaculum*	0
	b_Otu001032	*Acidobacteria*	*Gp10*	2
	b_Otu001102	*Chloroflexi*	*Litorilinea*	7
	f_Otu00170	*Glomeromycota*	*Glomeraceae*	5
	f_Otu00218	*Basidiomycota*	*Solicoccozyma*	2
	**f_Otu00260**	***Ascomycota***	***unclassified_Hypocreales***	**1**
Connectors	b_Otu000064	*Verrucomicrobia*	*Opitutus*	7
