# Supplementary material for: Linking belowground microbial network changes to different tolerance level towards Verticillium wilt of olive
Source: Microbiome. 2020 Feb 1;8:11. doi: 10.1186/s40168-020-0787-2 (PMC6995654; doi:10.1186/s40168-020-0787-2)

Figure S12. Co-occurrence networks of functional (RNA) communities from rhizosphere of both cultivars before and after inoculation.

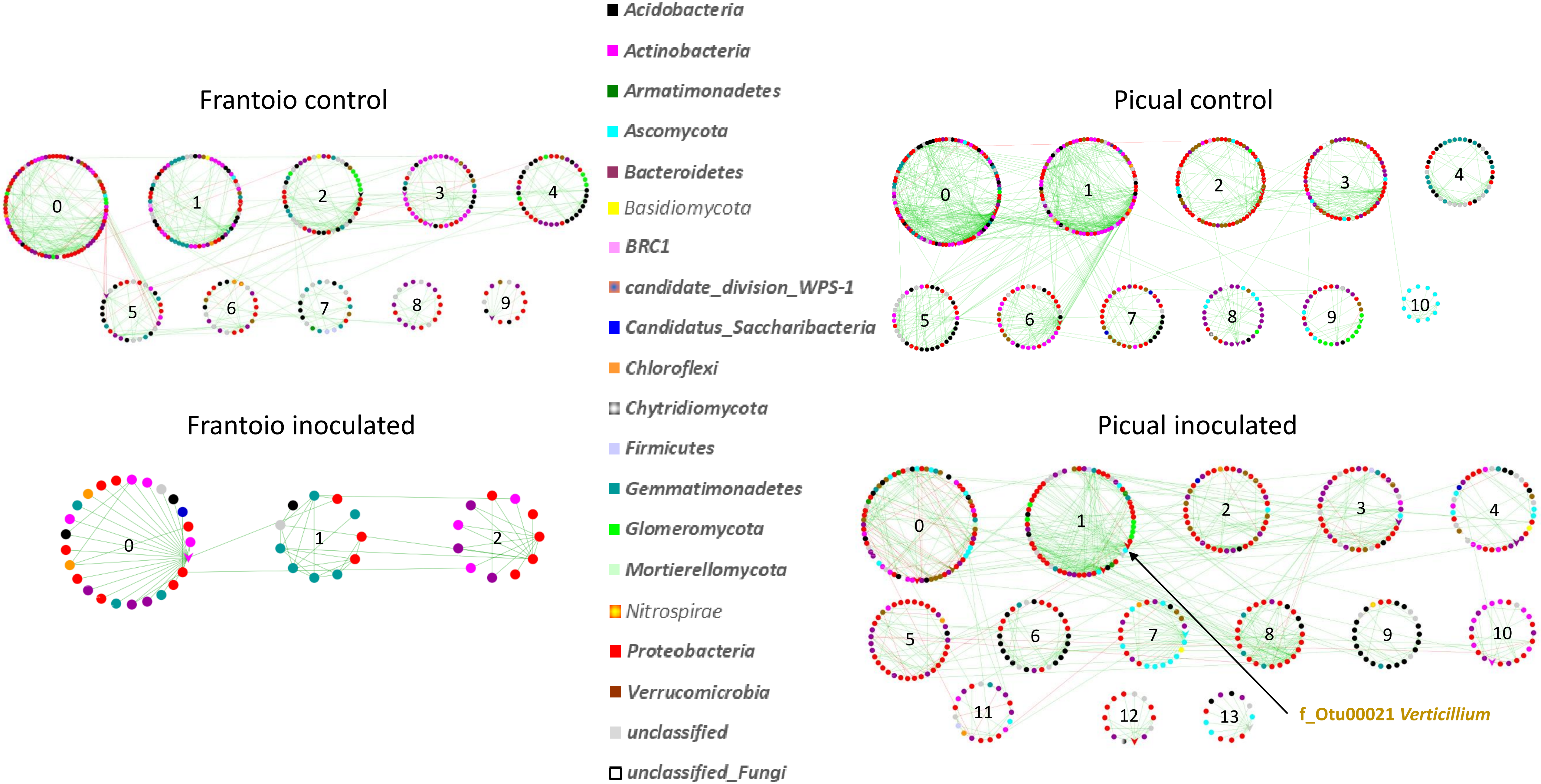

Supplement: Supplementary file 18 — Additional file 17: Figure S12. Co-occurrence networks of functional (RNA) communities from rhizosphere of both cultivars before and after inoculation. [file 40168_2020_787_MOESM17_ESM.pdf]
